# Runx2 stimulates neoangiogenesis through the Runt domain in melanoma

**DOI:** 10.1038/s41598-019-44552-1

**Published:** 2019-05-29

**Authors:** Daniela Cecconi, Jessica Brandi, Marcello Manfredi, Michela Serena, Luca Dalle Carbonare, Michela Deiana, Samuele Cheri, Francesca Parolini, Alberto Gandini, Giulia Marchetto, Giulio Innamorati, Francesco Avanzi, Franco Antoniazzi, Emilio Marengo, Natascia Tiso, Monica Mottes, Donato Zipeto, Maria Teresa Valenti

**Affiliations:** 1 Department of Biotechnology, Mass Spectrometry & Proteomics Lab, University of Verona, Verona, Italy; 2 Department of Sciences and Technological Innovation, University of Piemonte Orientale, Vercelli, Italy; 3 Department of Medicine, University of Verona, Verona, Italy; 4 Department of Neurosciences, Biomedicine and Movement Sciences, University of Verona, Verona, Italy; 5 Department of Surgery, Dentistry, Pediatrics and Gynecology, University of Verona, Verona, Italy; 6 Department of Biology, University of Padova, Padova, Italy

**Keywords:** Melanoma, Protein-protein interaction networks

## Abstract

Runx2 is a transcription factor involved in melanoma cell migration and proliferation. Here, we extended the analysis of Runt domain of Runx2 in melanoma cells to deepen understanding of the underlying mechanisms. By the CRISPR/Cas9 system we generated the Runt KO melanoma cells 3G8. Interestingly, the proteome analysis showed a specific protein signature of 3G8 cells related to apoptosis and migration, and pointed out the involvement of Runt domain in the neoangiogenesis process. Among the proteins implicated in angiogenesis we identified fatty acid synthase, chloride intracellular channel protein-4, heat shock protein beta-1, Rho guanine nucleotide exchange factor 1, D-3-phosphoglycerate dehydrogenase, myosin-1c and caveolin-1. Upon querying the TCGA provisional database for melanoma, the genes related to these proteins were found altered in 51.36% of total patients. In addition, VEGF gene expression was reduced in 3G8 as compared to A375 cells; and HUVEC co-cultured with 3G8 cells expressed lower levels of CD105 and CD31 neoangiogenetic markers. Furthermore, the tube formation assay revealed down-regulation of capillary-like structures in HUVEC co-cultured with 3G8 in comparison to those with A375 cells. These findings provide new insight into Runx2 molecular details which can be crucial to possibly propose it as an oncotarget of melanoma.

## Introduction

In the past half century, the incidence of metastatic melanoma has increased in the general population^1^. Western life style may possibly contribute to melanoma occurrence and the mortality rate associated to metastatic melanoma is very high. Different factors, endogenous or environmental, are associated to malignant transformation in melanoma. Among them, previous family cases of melanoma as well as many dysplastic nevi are strongly associated to melanoma^2^. In addition, exposure to sun or ultraviolet radiations, phenotype characterized by blond or red hair and pale skin, reduction of immune protection and chemical exposures contribute to melanoma onset^3–5^.

Several studies identified the molecular events involved in the malignant transformation of melanoma. Transcription regulators and proto-oncogenes such as serine/threonine kinase B-Raf (BRAF), mast/stem cell growth factor receptor c-Kit (KIT), neuroblastoma RAS viral oncogene homolog (NRAS), phosphatase and tensin homologue (PTEN), tumor suppressor p53 (P53), telomerase reverse transcriptase (TERT), and microphthalmia-associated transcription factor (MITF) are associated with metastatic melanoma. In addition, mutations in cyclin-dependent kinase inhibitor 2A (CDKN2A) encoding two tumour suppressor proteins, p16 and p14, as well as genes involved in the DNA repair mechanism or the melanocortin-1 receptor (MC1R) play an important role in the onset of melanoma^2,6^. We recently reported new melanoma oncotargets, such as enolase 1, parkinsonism-associated deglycase, prostaglandin E synthase 3, nucleophosmin, and stathmin 1, playing a key role in malignant transformation^7^. Moreover, molecular analyses showed the central role of the PTEN/mutated in multiple advanced cancers 1 (MMAC1) in reducing cell proliferation or inducing apoptosis^8^, and the association of PTEN loss to melanoma^9^. Furthermore, angiogenesis is an important factor allowing melanoma migration and metastases. Mehnert *et al*. reported that the expression of vascular endothelial growth factor (VEGF) and its receptors (VEGF-R) are higher in melanomas compare to normal melanocytes^10^.

Recently, we identified the involvement of the Runt domain of the Runt-related transcription factor 2 (RUNX2) gene in melanoma^11^. RUNX2 is the most important transcription factor involved in osteogenic differentiation. RUNX2 is also ectopically expressed in several solid tumors such as in thyroid, breast, pancreatic, prostate, lung, ovarian epithelial cancers^12,13^ and melanoma^14^. We reported that RUNX2 promotes migration and melanoma proliferation and restricts the apoptosis through its Runt domain^11^. Importantly, the Runt domain downregulates P53 expression as well as single-stranded DNA-binding protein 1 (SSBP1), a protein that controls the stability and the transcriptional activity of P53^15^.

Therefore, in order to identify the molecular pathways associated with RUNX2 in melanoma we performed a comparative shotgun proteomic analysis of Runt KO (3G8) and wild-type (A375) melanoma cells. We identified for the first time proteins associated with the Runt domain involved in promoting migration, proliferation and in particular in neoangiogenesis of melanoma cells. We then confirm the role of Runx2 in neoangiogenesis, analyzing the gene expression of VEGF in 3G8 and A375 cells, and performing co-culture experiments using human umbilical vein endothelial cell (HUVEC) plus 3G8 or A375 cells. We observed that HUVEC express lower levels of CD105 and CD31 neoangiogenetic markers and have reduced network-like structures when co-cultured with 3G8 cells, confirming a role for Runx2 in neoangiogenesis.

## Experimental Procedures

### Cell cultures and co-cultures

A375 melanoma cells (at passage 5) and normal human epidermal melanocytes (NHEM) were purchased from American Type Culture Collection (ATCC^©^ CTRL-1619^TM^) (Rockville, MD, USA). Cell lines were cultured under a humidified atmosphere of 5%CO_2_ and passage in growth medium: RMPI 1640 (Sigma-Aldrich) containing 10% FBS (fetal bovine serum) (Sigma-Aldrich; Merck Millipore) supplemented with antibiotics (1% penicillin/streptomycin) and 1% glutamine. Once 70–80% confluence was reached, cells were harvested using trypsin, washed and counted on a microscope using a Burker hemocytometer.

Primary HUVEC (at passage 10) were kindly provided from Prof. Bazzoni of the University of Verona. Cells were cultured in a 75 cm^2^ culture flask coated with Matrigel (Corning Incorporated; NY, USA) in the presence of M200 medium and 10% of LSGS supplement (Thermo Fisher scientific, Waltham, MA, USA) under a humidified atmosphere of 5% CO_2_. Co-cultures for CD105 gene and CD31 gene/protein expression analyses, as well as for net-like structure detection, were performed using 6 wells plate Transwell with 0.4 µm pores (Corning Incorporated; NY, USA). HUVEC cells were plated in the chamber, in presence of M200 supplemented medium, while in the permeable membrane were plated A375, 3G8 Crisped clone and NHEM cells using RPMI + 10% FBS. Each co-culture was performed in three replicates.

### CRISPR/Cas9

The CRISPR/Cas9 technique was applied as previously reported^11^. In brief, we used the following two specific gRNAs designed by analyzing the target sequence with both CHOPCHOP^16,17^ and MIT (http://crispr.mit.edu/) CRISPR design tools (gRNA A CCCATCTGGTACCTCTCCGA; gRNA B GATCGTTGAACCTTGCTACT). The above gRNAs were individually cloned in the PX459 V2.0 Cas9 expressing vector (Addgene), following the protocol described by Ran *et al*.^18^. The A375 cells were co-transfected and single cell clonings were obtained as previously reported^11^. To analyse the effects of CRISPR/Cas9 editing we called the WT and CRISPR/Cas9 edited cell lines A375 WT and 3G8, respectively. To confirm the deletion, we sequenced the PCR products by Sanger Sequencing. In particular, the specific Runt domain was amplified by PCR with the following primers: FW TGAAGTGGCATCACAACCCA; RV AGTCAGAGACCTACCTCGTC. The PCR products were purified with the FastGene® extraction kit (Nippon Genetics, Tokyo, Japan) and the forward PCR primer was used for Sanger sequencing (GenomeLab™ DTCS quick start kit and CEQ. 8000 Genetic Analysis System, SCIEX, San Francisco CA, USA) following manufacturer’s instructions.

### Sample preparation for proteomics

A375 wild-type (WT) and 3G8 melanoma cells were collected, washed in 1x phosphate-buffered saline (PBS), resuspended in RIPA buffer (50 mM Tris-HCl pH 8.0 with 150 mM NaCl, 1.0% Igepal CA-630, 0.5% sodium deoxycholate and 0.1% sodium dodecyl sulfate, Sigma-Aldrich) supplemented with a protease inhibitors cocktail 1x (Roche) and sonicated for 5–6 cycles. The lysate was gently mixed for 15 min and then centrifuged at 14,000 × g for 15 min at 4 °C to pellet cell debris. The supernatant was collected and total protein concentration was determined using a bicinchoninic acid protein assay (Sigma-Aldrich). Before mass spectrometry (MS) analysis, proteins were digested by trypsin following the protocol previously described^19^.

### Mass spectrometric analysis and data processing

Protein identification and quantification by liquid chromatography-tandem mass spectrometry (LC-MS/MS) analyses were performed as previously reported^19^. Briefly, LC–MS/MS analyses were performed using a micro-LC Eksigent Technologies (Dublin, USA) system interfaced with a 5600 + TripleTOF system (AB Sciex, Concord, Canada). The injection volume of each sample was 4.0 μL. Samples used to generate the Sequential Window Acquisition of All Theoretical Mass Spectra (SWATH-MS) spectral library were subjected to the traditional data-dependent acquisition (DDA), and then to cyclic data independent analysis (DIA) of the mass spectra, using a 25-Da window. The MS data were acquired with Analyst TF 1.7 (AB SCIEX, Concord, Canada). Three instrumental replicates for each sample were subjected to the DIA analysis. The MS files were searched for protein identification. PeakView 1.2.0.3 and Protein Pilot software v. 4.2 (AB SCIEX, Concord, Canada) were used to generate the peak-list. The mass spectrometry files were searched using Protein Pilot (AB SCIEX, Concord, Canada), Mascot (Matrix Science, Inc, Boston, MA).

Samples were input in the Protein Pilot software v. 4.2 (AB SCIEX, Concord, Canada), which employs the Paragon algorithm, with the following parameters: cysteine alkylation, digestion by trypsin, no special factors and False Discovery Rate (FDR) at 1%. The UniProt Swiss‐Prot reviewed database containing human proteins (version 2015.07.07, containing 42131 sequence entries). The Mascot search was performed on Mascot v. 2.4, the digestion enzyme selected was trypsin, with two missed cleavages and a search tolerance of 50 ppm was specified for the peptide mass tolerance, and 0.1 Da for the MS/MS tolerance. The charges of the peptides to search for were set to 2+, 3+, and 4+, and the search was set on monoisotopic mass. The instrument was set to ESI‐QUAD‐TOF and the following modifications were specified for the search: carbamidomethyl cysteines as fixed modification and oxidized methionine as variable modification.

Quantification was performed by integrating the extracted ion chromatogram of all the unique ions for a given peptide. SwathXtend was employed to build an integrated assay library with the DDA acquisitions, using a protein FDR threshold of 1%. Quantification was carried out with PeakView 2.0 and MarkerView 1.2. (ABSCIEX, Concord, Canada). The six peptides per protein with the highest MS1 intensity and six transitions per peptide were extracted from the SWATH files. Shared peptides were excluded as well as peptides with modifications. Peptides with FDR lower than 1.0% were exported in MarkerView for the t-test. The up- and down-regulated proteins were selected using p-value < 0.05 and fold change >1.5.

### Western blotting

Western blotting was performed as described in our former study^20^. Briefly, protein samples from two biological replicates were diluted 1:1 in Laemmli’s sample buffer (62.5 mM Tris-HCl, pH 6.8, 25% glycerol, 2% SDS, 0.01% Bromophenol Blue), heated for 5 min at 90 °C, and separated by sodium dodecyl sulfate−polyacrylamide gel electrophoresis (SDS-PAGE), followed by transfer onto polyvinylidene difluoride (PVDF) membranes. Amido Black staining (Sigma-Aldrich) was used to confirm equal protein loading in different lanes. Then the primary and secondary antibodies were incubated with PVDF membranes (Supplemental Table 1), and chemiluminescent signal acquisitions were done with the ChemiDoc MP Imager, a CCD imager, using the Image Lab 5.2.1 software (Biorad).

### Bioinformatics analysis

Identified proteins were annotated according to the information associated to their main functions available under gene ontology (GO) categories using the Database for Annotation, Visualization and Integrated Discovery (DAVID) (v6.8) (http://david.abcc.ncifcrf.gov/)^21^. Functional annotation chart report was used to identify GO biological processes, molecular function and cellular component. Gene enrichment analysis was performed by setting the threshold of EASE Score, a modified Fisher Exact P-Value, to 0.1 and p-value smaller than 0.01, while the threshold of minimum gene counts belonging to an annotation term was set to 2.

Moreover, Ingenuity Pathway Analysis (IPA, Ingenuity Systems, Redwood City, CA) was performed, as previously described^22^, in order to identify the perturbed canonical pathways associated with deregulated proteins of 3G8 cells. Briefly, proteins associated with canonical pathways were estimated as significant using the Fisher’s exact test (p-value ≤ 0.01). In order to further explain the observed expression changes in 3G8 cells, the IPA Upstream Regulator analysis was performed to identify a putative cascade of upstream regulators. The upstream regulators were assumed as valid effectors of gene/protein expression if the corresponding p-value obtained by Fisher’s exact test was ≤0.01. Activation z-score algorithm was used to allow for prediction whether an upstream regulator is activated (z ≥ 2) or inactivated (z ≤ −2) based on the direction of expressional change of the associated genes.

Finally, protein-protein interaction network analysis was carried out using STRING platform (http://string-db.org)^23^ by setting the specie under investigation (Homo sapiens) and medium confidence level (score 0.4). We retrieved known interactions experimentally determined (pink edge) and on curated database (light blue edge), excluding all other prediction methods implemented in STRING (such as co-expression and text-mining). Additional white nodes and network depth were kept to the minimum value^1^, to exclude as many false positive interactions as possible. Genes were subjected to k-means clustering for five clusters and disconnected nodes were excluded.

The Cancer Genome Atlas (TCGA) of skin cutaneous melanoma dataset that consisted of the maximum number of sequenced cases (471 patients) was interrogated through the cBio Cancer Genomics Portal (http://www.cbioportal.org/) for alterations in our query list consisting in proteins associated to angiogenesis, i.e. fatty acid synthase (FASN), chloride intracellular channel protein-4 (CLIC4), heat shock protein beta-1 (HSPB1), Rho guanine nucleotide exchange factor 1 (ARHGEF1), D-3-phosphoglycerate dehydrogenase (PHGDH), myosin-1c (MYO1C), and caveolin-1 (CAV1) for missense, truncating and in-frame mutations, amplification and deletions, mRNA up- and downregulation and protein up- and down regulation by reverse phase protein array (RPPA) assay. A survival analysis was also carried out comparing the cases with alterations in all, or in each, of the proteins involved in angiogenesis to the ones without alterations to look at the median overall survival.

### Real time RT-PCR

Total RNA extraction and reverse transcription were performed as previously reported^11^. PCRs were performed in a total volume of 25 µl using 20 ng of cDNA from each sample. For endoglin (CD105, Hs00923996_m1), platelet endothelial cell adhesion molecule (CD31, Hs1065279_m1), and VEGF-A (Hs00900055_m1) expression analysis, we used TaqMan® probes (Life Technologies LTD, CA, USA) and TaqMan® Universal Master Mix (Life Technologies LTD, CA, USA). Gene expression was normalized to housekeeping Beta-2 Microglobulin (B2M, Hs99999901_s1) gene, calculating the relative fold expression differences. TaqMan SDS analysis software was used to analyse the Ct values. Three independent experiments with three replicates for each sample were performed.

### Immunofluorescence

After 7 days of co-culture, cells were fixed and processed according to the manufacturer’s protocols. As primary antibody we used CD31 (Dako, Glostrup, Denmark), diluted 1:20 in Antibody Dilution Buffer and incubated 2 hours at room temperature. Slides were then incubated with goat mouse fluorescein conjugated (Cat. AP124F, Millipore) secondary antibody for 2 hours at room temperature. Nuclear staining was performed by ProLong™ Gold Antifade Mountant with DAPI. Images were recorded using a EVOS Cell Imaging Systems Leica (Thermo Fisher scientific, Waltham, MA, USA) at 4X and 10X.

### Net-like formation assay

Six-wells Transwell plates, prepared as previously described, were evaluated after 7 days. The membrane was removed and HUVEC cells, plated in the chamber, were photographed under polarized phase at 10x magnification through a Leica DMi1 Microscope (Leica, Wetzlar, Germany). Completely enclosed spaces were counted to quantify net-like structures^24^.

### Capillary-like tube formation on matrigel-3D matrix

Capillaries formation was evaluated in Matrigel (Corning, NY, US) according to manufacturer’s instructions. Briefly, 100 µl of Matrigel (5 mg/mL) were aliquoted in each 24 well plate and incubated at 37 °C for 30 minutes. After trypsinization 4.80 × 10^5^/mL of HUVEC were suspended in 270 μL Matrigel matrix solution (5 mg/mL) and incubated at 37 °C for 30 to 45 min. After 12 hours 24 well transwell inserts (Sarsted, Nümbrecht Germany) were added to the HUVEC seeded plate and incubated 37 °C. After 24 hours of co-culture incubation under normoxia, tubes formation was observed using DMi1 microscope (Leica, Wetzlar, Germany) at 5x and 10x magnifications.

### GFP V2a kit tube formation assay

Tube formation was also evaluated by a Matrigel-green fluorescent protein (GFP) fluorescence coupling system. 24 well plate was coated using 5 mg/mL of Matrigel (Corning, NY, US) and incubated at 37 °C for 30 minutes. HUVEC-GFP cells were plated according to the datasheet instructions and after 24 hours 24 well transwell inserts (Sarsted, Nümbrecht Germany) were co-cultured for 24 hours. Tubes formation was observed under Evos FL auto microscope (Leica, Wetzlar, Germany) using a GFP channel at 4x and 10x magnifications and the tubular structures formed in the matrigel were counted in 5 random fields.

### Experimental design and statistical rationale

The SWATH-MS-based proteomics analysis was performed on a total number of two biological replicates (obtained by seeding and growing the cells in separate times) for both 2G8 cells and control A375 cells. Three instrumental replicates for each sample were then subjected to the DIA analysis. Co-culture experiments with HUVEC cells, as well as RT PCR analysis, were performed in three independent experiments with three replicates for each sample.

We used the Student Paired t-test to compare the variation of a variable between two groups, while one-way analysis of variance (ANOVA) was used for multiple comparisons. Differences were considered statistically significant with p < 0.05. Statistical analyses were performed using SPSS for Windows, version 16.0 (SPSS Inc, Chicago, IL, USA).

## Results

### Deletion of the Runt domain in A375 melanoma cells

**Figure 1:**
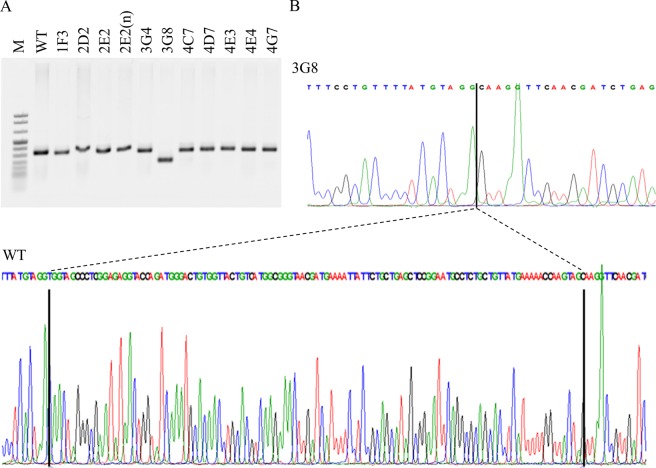
Deletion of the Runt domain in A375 melanoma cells. (**A**) PCR products. 11 clones have been generated by applying the CRISPR/Cas9 technique. The PCR products, analysed by agarose gel electrophoresis, have been obtained by amplifying the Runt domain region. (**B**) Sanger sequencing chromatogram. The image shows the comparison between the WT and the 3G8 sequences with the deletion in genomic context.

After applying the CRISPR/Cas9 procedure to A375 cells, we performed a single cloning and expansion. Remarkably we obtained only 11 clones. Therefore, we analysed the sequences of the PCR products obtained by amplifying the Runt domain region (Fig. 1A).

**Figure 2:**
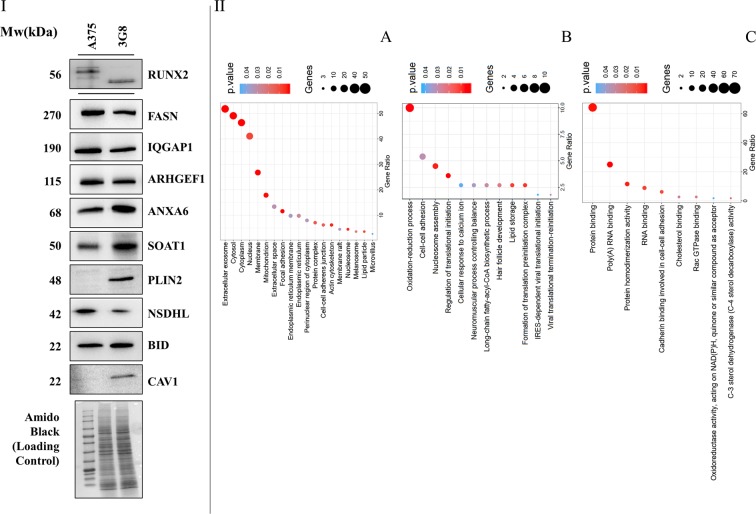
Runt domain-dependent effects on the proteome of melanoma cells. (**I**) Immunoblot analysis of Runx2 and of some deregulated proteins (indicated by the corresponding gene name) in A375 and 3G8 cells. The primary antibodies, along with the sources and working dilutions are listed in Supplementary Table 1. The blots were cropped to focus upon the specific proteins indicated. Amido black-stained PVDF membrane is also presented to show equal loading of protein. (**II**) Functional annotation of 3G8 deregulated proteins according to DAVID. The enriched cellular component (**a**), biological process (**b**), and molecular function (**c**) terms are reported.

As the sequence in 10 of 11 clones showed none or little changes, such as in-frame insertion or deletion (Supplementary Fig. 1) compared to A375, we chose to investigate the clone 3G8 in which a deletion corresponding to 114 bp was observed (Fig. 1B). Before further experiments, we confirmed by immunoblotting in 3G8 cells the expression of the remaining protein at comparable level of the Runx2 full length (Fig. 2I), as we previously published^11^.

### Quantitative analysis of Runt domain-dependent effects on the proteome of melanoma cells

To assess the global effects of Runt domain deletion from RUNX2, a comparative shotgun proteomic analysis of A375 and 3G8 melanoma cells was carried out on two biological replicates. We identified a total of 1809 proteins in the first biological replicate, and 1671 in the second one, with 1445 identified proteins shared in common. In total 1169 protein groups were quantified in the two biological replicates with a peptide confidence cut-off of 99% (FDR < 1%); among these 112 were deregulated. In particular, 41 proteins were found to be up-regulated in 3G8 melanoma cells compared to controls, while 71 were found down-regulated (p < 0.05, fold-change cut-off > 1.5). The lists of: “identified peptides and their area”, “identified proteins and their normalized area”, “quantified and differentially expressed proteins” are presented in Supplementary Tables 2, 3, 4 respectively.

To validate proteomic data, we performed immunoblot analysis to determine the relative amount of up and down-regulated candidates in 3G8 cells compared to A375 cells (Fig. 2I).

These proteins were selected on the basis of several factors, such as fold of variation, novelty and relevance. The results of immunoblot experiments were largely consistent with those observed from the quantitative proteome analysis.

### Modulation of biological processes and pathways induced by inhibition of Runx2 DNA-binding function in melanoma cells

To reveal signalling pathways and interaction networks perturbed by inhibition of Runx2 DNA-binding function, we performed a bioinformatics analysis on the proteins whose expression significantly changed between A375 and 3G8 melanoma cells.

Cellular component analysis annotated more than half of differentially expressed 3G8 proteins to the extracellular exosome (51,8%), followed by a smaller percentage of annotated protein to cytosol localization (49,1%) (Fig. 2IIa). In addition, the GO enrichment analysis of biological process suggested that the deregulated 3G8 proteins have a role in oxidation-reduction (10%), cell-cell adhesion (5,4%) and nucleosome assembly (4,5%) (Fig. 2IIb). According to the molecular function analysis, most of the deregulated proteins in 3G8 cells exhibited “binding” activity, in particular protein (64,3%) and poly(A) RNA (25%) binding activity (Fig. 2IIc).

The complete list of GO terms for cellular component, biological process and molecular function analysis, together with p-value and genes, is reported in Supplemental Table 5.

**Figure 3:**
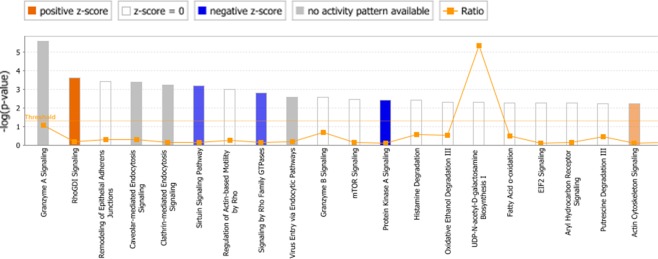
Identification of deregulated canonical pathways in 3G8 cells based on IPA. The top 20 significantly altered canonical pathways associated with deregulated proteins of 3G8 cells. The y-axis corresponds to the −log of the P-value (Fisher’s exact test) and the ratio (orange points) the number of genes in a given pathway that meet cut off criteria, divided by the total number of genes that map to that pathway. The orange and blue colored bars indicate predicted pathway activation, and predicted inhibition, respectively (z-score). White bars are those with a z-score at or very close to 0. Gray bars indicate pathways where no prediction can currently be made.

**Figure 4:**
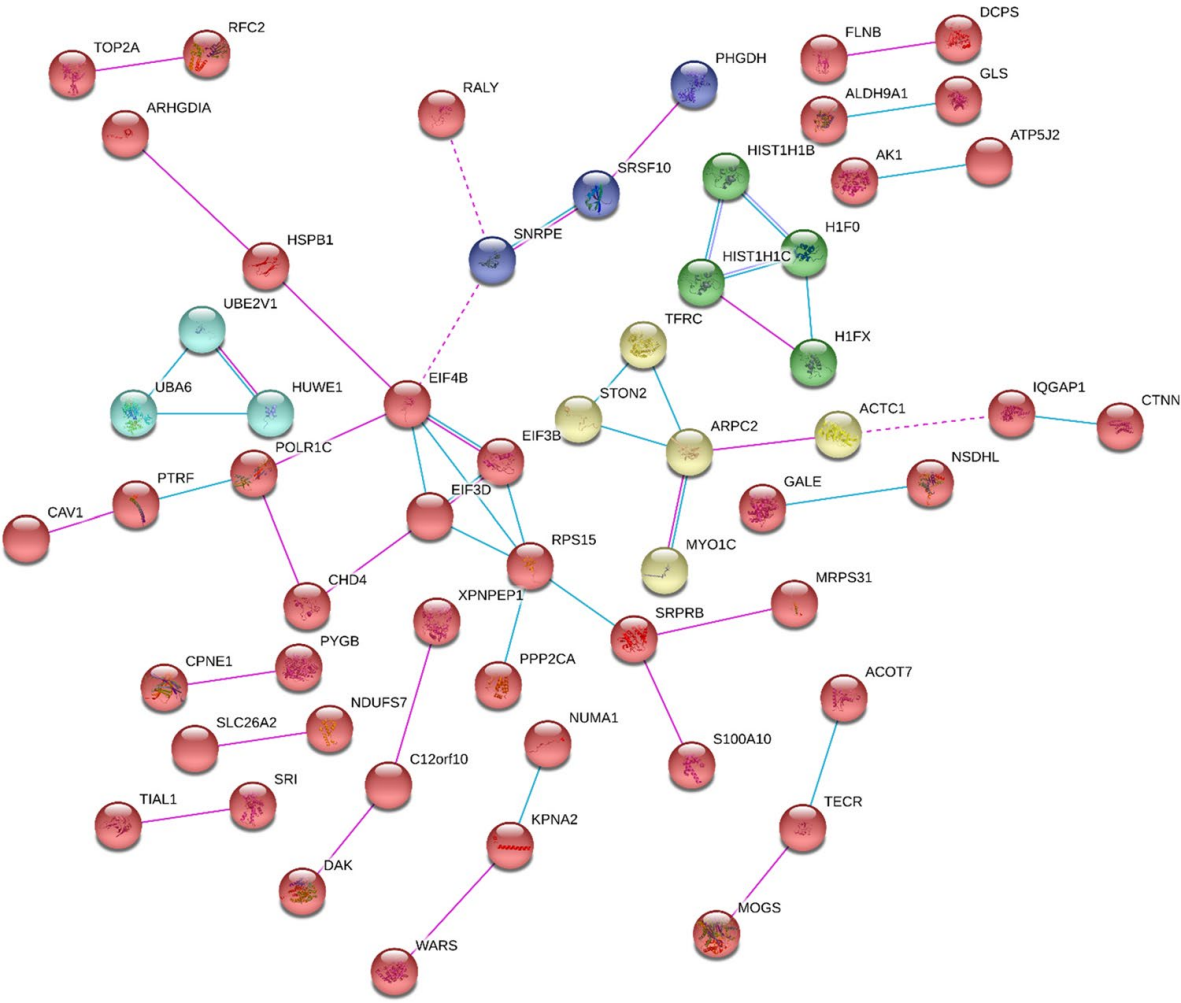
Protein network of modulated proteins in 3G8 cells by using STRING. Schematic view of known and predicted protein-protein interactions according to the STRING database (v. 10). The minimum required interaction score was set as the medium confidence (0.400) and the disconnected nodes in the network were hidden. Pink edges represent interactions experimentally determined, while light blue edges represent known interaction from curated database. The network map was clustered in 5 clusters using the K-means method

To reveal functional enrichment and deregulated pathways within the proteins that exhibited a changed abundance in 3G8 cells we performed an IPA analysis (Supplemental Table 6). Overall, IPA software revealed that proteins modulated in 3G8 cells show a significant link with many different pathways. The twenty top canonical pathways (p-value ≤ 0.001) are shown in Fig. 3. Among them, the “Granzyme A signalling” is the most statistically significant one. Notably, an overall increase in the activity of “Rho GDI signalling” and “actin cytoskeleton pathways” was detected (orange histograms), in addition to an overall decrease in the activity of “sirtuin”, “Rho GTPases” and “protein kinase A” signalling pathways (blue histograms). Moreover, the IPA software allowed us to examine also the potential upstream regulators associated with the above-described proteomic profile. Interestingly, the most significant upstream regulators include the transforming growth factor beta 1 (TGFB1; p = 7.65E-09, z = 0.148), tretinoin (p = 6.24E-07, z = 1.437) and mir-122 (p = 5.98E-06, z = 1.044), as well as the ligand-dependent nuclear estrogen receptor (ESR1; p = 8.30E-05, z = 0.289), the transcription regulators N-myc (MYCN; p = 8.86E-05, z = −1.39) and tumor protein p53 (TP53; p = 1.12E-04, z = 0.218). In addition, the transcription regulator retinoblastoma (RB1; p = 3.40E-03, z = −2.05) is predicted to be significantly inhibited. A complete list of transcriptional upstream regulators with significant p-values (p ≤ 0.01) can be found in Supplemental Table 6. To determine the key proteins in the function network, the STRING online tool was used to analyse the protein-protein interaction networks. The analysis resulted in a network (showed in Fig. 4) with an average local clustering coefficient (i.e. indication of the embeddedness of single nodes) of 0.374, and an average node degree (i.e. number of interactions, at the score threshold, that a protein has on the average in the network) of 0.9. The low average local clustering coefficient suggests the presence of low connected neighborhoods in the network, as indicated by the presence of small groups of interacting proteins (2 or 3 proteins). The results showed that 5 clusters were determined by the K-means method, represented by different colored nodes in Fig. 4, with a maximum and minimum size of 42 and 3 genes respectively. In particular, the green cluster has 4 members and was found to be enriched in histone subunits, the yellow cluster has 5 members 3 of which are components of the cytoskeleton, while the light-blue cluster has 3 members which play a role in ubiquitin pathway

### Runt domain increases angiogenesis ability in melanoma cells

**Figure 5:**
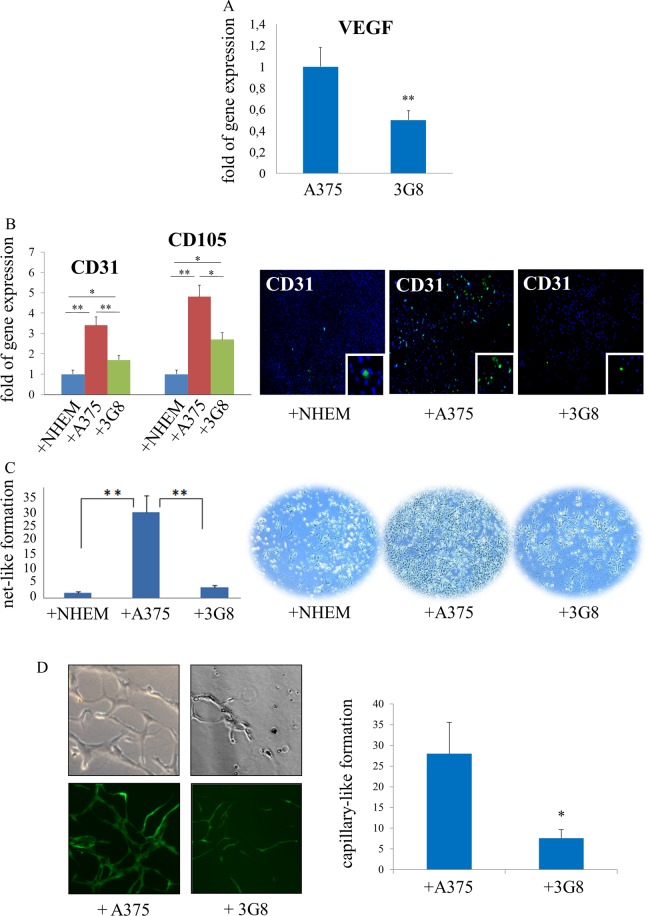
Runt domain promotes angiogenesis in melanoma cells. (**A**) Real-Time PCR analysis shows decreased gene expression of VEGF in 3G8 melanoma cells as compared to A375 control cells. (**B**) Real-time PCR (left) and immunofluorescence (right) respectively show significant down-regulation of CD31 and CD105 mRNAs and reduced CD31 protein expression in HUVEC cells co-cultured with 3G8 cells compared to that co-cultured with A375 cells. (**C**) In the transwell co-culture setup HUVEC cells were cultured as a monolayer in the chamber and NHEM, A375, or 3G8 cells were plated in the upper well. Quantification (left) and representative images (right) of the tube formation assay are shown; magnification 10x. (**D**) Representative phase contrast and epifluorescence images of capillary-like structure formation in HUVEC (upper panels) and HUVEC-GFP (bottom panels) co-cultured with A375 or 3G8 cells on matrigel-3D matrix; magnification 40x (left). The branch points of the capillary-like tubes were counted and the quantitative data are presented (right). Statistical significance was calculated based on three independent experiments (*p < 0.05; **p < 0.01, p-values between depicted groups). NHEM = normal melanocytes.

In order to prove the involvement of Runt domain in inducing neoangiogenesis, as suggested by the proteomic analysis, we analysed the expression of VEGF gene in A375 and 3G8 melanoma cells. Interestingly, as shown in Fig. 5A we found a downregulation of VEGF expression in 3G8 cells (p < 0.01) as compared to A375 cells. Therefore, we investigated the expression of CD105 and CD31, the markers of tumour neoangiogenesis, in HUVEC co-cultured with NHEM, A375 or 3G8 melanoma cells. The data obtained showed that HUVEC cells express lower levels of CD105 and CD31 mRNAs and reduced expression of CD31 protein when co-cultured with 3G8 as compared to A375 cells (Fig. 5B).

Moreover, further confirming the role of Runt domain in neoangiogenesis, we detected by transwell co-culture setup a reduced number of network-like structures in HUVEC co-cultured with 3G8 (p < 0.01, Fig. 5C) as compared to A375 cells. The pro-angiogenic effect of Runt domain was also assessed by capillary-like tube formation assays in the matrigel-3D matrix. We observed that HUVEC co-cultured with 3G8 cells had a significant reduction in the capillary forming features with few connecting structures when compared with A375 cells (p < 0.05, Fig. 5D). All these findings support the role of the Runt domain in promoting the neoangiogenesis.

### TCGA analysis indicates that angiogenic proteins modulated in 3G8 cells are altered in melanoma patients

**Figure 6:**
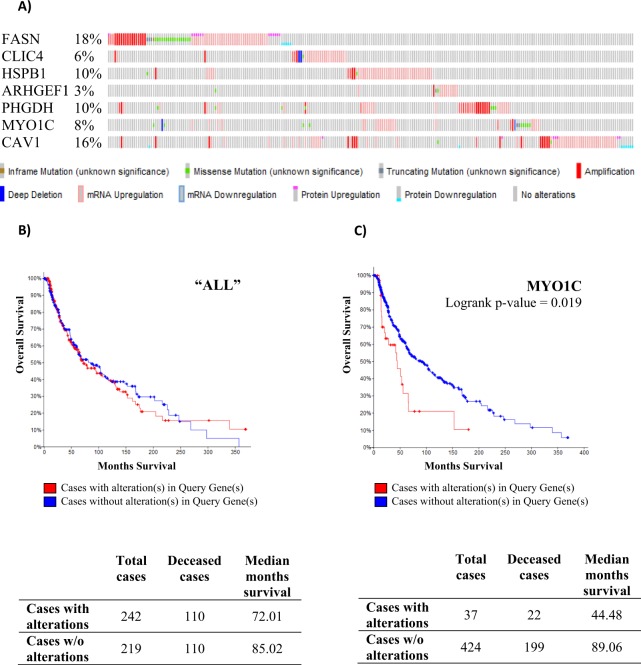
cBioPortal query of the TCGA provisional database for skin cutaneous melanoma dataset for alterations in genes of the list of targets implicated in angiogenesis (i.e. FASN, CLIC4, HSPB1, ARHGEF1, PHGDH, MYO1C, CAV1) and correlation with overall survival. (**A**) Combined alterations of 7 genes for our assay targets were seen in 242 cases (51.36%) of melanoma in the TCGA dataset. (**B**) Patients with alterations in these genes had no a statistically significant lower survival when compared to cases with no alterations, while (**C**) patients with alterations in MYO1C gene had a statistically significant lower survival when compared to cases with no alteration.

Upon querying the TCGA provisional database for skin cutaneous melanoma, consisting of 471 patients, our results indicate that our target list of proteins associated to angiogenesis (that depend on Runt domain) was found to be altered in 51.36% of the total patients (Fig. 6A). In particular, FASN was found altered in 17.95% of patients, while CLIC4, HSPB1, ARHGEF1, PHGDH, MYO1C, CAV1 were found altered in 6.05%, 9.6%, 2.92%, 9.6%, 7.72%, 15.87% of patients respectively. Using TCGA cohort survival data in univariate analysis, alterations in all these genes were not associated with reduced patient overall survival (Fig. 6B), while there was significantly lower overall survival among melanoma patients with alterations of MYO1C gene (Logrank test p-value: 0.0019) (Fig. 6C).

## Discussion

Our previous study showed that Runt domain KO melanoma cells are characterized by reduced proliferation, epithelial-mesenchymal transition and metastasis, suggesting that the Runt domain of Runx2 may represent an oncotarget in melanoma^11^. In the present study, with the aim of deepening the understanding of Runt domain-dependent effects on melanoma cells biology, we carried out a proteomic analysis of A375 and del-Runt (3G8) melanoma whole-cell extracts to investigate the intracellular molecular mechanisms characterizing the deletion of the DNA-binding domain from RUNX2.

The data obtained revealed the regulation of some key pathways in 3G8 cells. One of the interesting observations was that the most significant deregulated pathways in cells with the deletion of Runt domain of Runx2 is the “Granzyme A signalling”. The identified proteins of 3G8 cells involved in this signalling are all downregulated histone subunits (Supplemental Table 6) which are typical targets of granzyme A degradation^25^. Granzyme A is a selective protease that triggers a form of cell death characterized by all of the morphological features associated with apoptosis (membrane blebbing, chromatin condensation and nuclear fragmentation) without activating the caspase cascade. Accordingly, in our previous study, we observed an increased apoptosis in Runt domain KO cells; therefore, the downregulation of this protease corroborates the negative role of the Runt domain in inducing apoptosis in melanoma cells. Interestingly, it has been reported that Runx3 regulates the expression of granzyme B^26^, and that RUNX2^fl/fl^ mice have defects in granzyme B expression^27^. To the best of our knowledge, our finding represents the first indication that a link between Runx2 and Granzyme A may also exist. Consistent with our previous study^11^ the other data obtained by canonical pathway enrichment analysis confirmed the effect of the Runt domain of Runx2 on cell migration, metastasis and proliferation of tumour cell. Indeed, among the top enriched and induced pathways we found the “signalling of RhoGDI” a guanine nucleotide dissociation inhibitor (GDI) specific for the “Rho family of small GTPases”, a signalling instead detected as inhibited in 3G8 cells. Emerging evidences suggests that RhoGDI (activated in 3G8 cells) plays dual opposite roles in tumour progression^28^, while Rho GTPases (inhibited in 3G8 cells) act as promoters of cell migration^29^. Also the “sirtuin signalling pathways” which promotes melanoma metastases^30^, and the “protein kinase A signalling” implicated in the initiation and progression of tumours^31^, resulted to be inhibited pathways in 3G8 cells. In particular, among the 3G8 cells deregulated proteins that we identified by MS and immunoblotting analyses, there are the pro-apoptotic BH3-interacting domain death agonist (BID, +1.84 fold change), as well as Ras GTPase-activating-like protein (IQGAP1, −1.54 fold change) which is involved in melanoma cell migration^32^, and annexin-VI (ANXA6, +1.93 fold change) which acts as a multifunctional scaffold in cell motility^33^ and represents a tumour suppressor in melanoma^34^.

Interesting data were also suggested by the upstream regulator analysis performed *in silico* by IPA (Supplemental Table 6). This analysis indicated that the most significant upstream regulators is TGF-β1, of which Runx2 represents one of the major targets^35^. We found interesting that the modulation of some of the TGF-β1 targets identified in this study, such as FASN, ANPEP, CLIC4, HSPB1, ARHGEF2, PHGDH, MYO1C and CAV1, are involved in processes that counteract angiogenesis. Interestingly, the involvement of Runx2 in regulation of angiogenesis and vasculogenic factors (such as VEGF) has already been demonstrated during bone development^36^ as well as in cancer^37–41^, but never in melanoma.

Notably, TGF-β1 markedly decreases the expression of fatty acid synthase^42^ which we found downregulated in 3G8 cells (−1.95 fold change). This enzyme besides playing a pivotal role in lipid metabolism, is also implicated in the induction of angiogenesis^43^, in particular in cancer cells^44^. Accordingly, FASN inhibitors play a key role in antiangiogenic treatments^45^; in particular, they reduce the tumour cell-mediated formation of HUVEC capillary-like structures in melanoma^46^. Accordingly, we found a reduced number of network-like structures in HUVEC cocultured with 3G8 compared to cocultures with WT melanoma cells (Fig. 5C). We also assed HUVEC capillary-like tube formation when in 3D co-culture with 3G8 or A375 cells using both a Matrigel (which provides similar conditions to that observed in the tumour microenvironment) as well as a Matrigel-GFP fluorescence coupling system (which allowed direct visual evaluation of pro-angiogenic effect of melanoma cells via comparing fluorescent tubular structures formed by GFP-expressing HUVECs, while obviating noise effect resulting from any nonspecific cell attachment to HUVEC targets). As depicted in Fig. 5D the formation of capillary-like structures on 3D-matrix was significantly reduced in HUVEC cells co-cultured with 3G8 as compared to A375 cells. Among the downregulated TGF-β1 targets that we identified in 3G8 cells there is also aminopeptidase N (ANPEP/CD13, −1.59 fold change) a ubiquitously expressed membrane peptidase. ANPEP is involved in melanoma angiogenesis and in melanoma cell invasion^47^. In particular, the inhibition of ANPEP in melanoma cells correlates with the anti-tumour angiogenesis effect of bestatin^48^. ANPEP is also an important mediator of resistance to inhibition of BRAF, one of the most aggressive oncogenes found in melanoma which modulates angiogenesis^49^. The proteomic analysis also suggested the downregulation of chloride intracellular channel protein-4 (CLIC4, −1.82 fold change), a p53 and TGF-β regulated protein which promotes angiogenesis supporting acidification of vacuoles along the intracellular tubulogenic pathway^50^ and induces tubular morphogenesis^51^. Emerging evidences indicate that targeting CLIC4 could represents a strategy to diminish some of the tumour enhancing effects of the cancer stroma^52^ and could further suppress the invasion in melanoma cells^53^. We also showed the downregulation in 3G8 cells of heat shock protein beta-1 (HSPB1, −1.55 fold change), a molecular chaperone highly expressed in many cancers^54^. It has been reported that by combining HSPB1 silencing and BRAF inhibition melanoma cells are fully committed to death^55^ and, accordingly, reduced level of HSPB1 correlates with a less aggressive phenotype and improved survival in patients with melanoma^56^. Interestingly, the downregulation of HSPB1 is associated to reduced endothelial cell proliferation and decreased secretion of key mediator of angiogenesis (such as VEGF-A, VEGF-C, and basic fibroblast growth factor)^56^. Accordingly, we observed a lower gene expression of VEGF-A in KO Runt cells (3G8) as compared to A375 melanoma cells (Fig. 5A). We also detected a reduced number of network-like structures, as well as reduced expression of CD105 and CD31 endothelial markers in HUVEC cells co-cultured with 3G8 as compared to that co-cultured with A375 melanoma cells (Fig. 5B,C). CD105 and CD31 are angiogenesis-associated molecules and they are considered direct markers of the levels of neoangiogenesis. In addition, CD105 and CD31 expression indicates the density of intra-tumorous vessels^57^ and it is considered important prognostic factor in cancer^57,58^, Therefore, our results strongly suggest that Runt domain promotes the neoangiogenesis in melanoma.

We also showed in 3G8 cells downregulation of Rho guanine nucleotide exchange factor 1 (ARHGEF1, −1.50 fold change) and 2 (ARHGEF2, −2.70 fold change) which are fine-tuners of angiogenesis and vascular function^59^; D-3-phosphoglycerate dehydrogenase (PHGDH, −1.89 fold change), a key enzyme of the serine synthesis pathway whose knockdown inhibits angiogenesis *in vitro* and *in vivo* (as a result of decreased nucleotide and heme synthesis)^60^; as well as of unconventional myosin-Ic (MYO1C, −1.81 fold change) whose depletion resulted in an anti-angiogenic effect due to reduced cell-surface and total cellular level of VEGFR2 the predominant receptor in angiogenic signalling^61^. Finally, we also found upregulation of Caveolin-1 (CAV1, +2.11 fold change) as a result of the deletion of the Runt domain of Runx-2 in 3G8 cells. CAV1 has a dual role in melanoma^62^: in advanced stages its expression is associated with enhanced metastatic potential, while, at earlier stages, it functions as a tumour suppressor^63^. Interestingly, some researchers showed a relationship between its induction and reduced angiogenesis^64^. Of note, we also checked cBioPortal and found that these proteins related to angiogenesis (i.e. FASN, CLIC4, HSPB1, ARHGEF1, PHGDH, MYO1C, CAV1) are altered in a total of 242 melanoma patients out of a total of 471 (51.36%). The most common alteration found in patients for all these targets is the mRNA transcriptional upregulation of their encoding genes, hence the fact that the deletions of Runt domain leads to a downregulation at protein level for all of them (with the exception of CAV1 which is upregulated) seems particularly interesting in confirming the key role of Runx2 in the development of melanoma. Moreover, by using cBio Cancer Genomics Portal, the overall survival rates of patients with melanoma were compared between tumour samples with or without alterations in each and in all these proteins. However, the results indicate that our list of proteins involved in angiogenesis, whose expression is connected to Runt domain, do not constitute a “signature” to predict survival of patients with melanoma.

Our approach provides a comprehensive view of molecular details that depend on Runx2 DNA binding function in melanoma cells. It should however be emphasized that the findings here reported are related to the consequences of deleting the Runt domain of Runx2, and not to Runx2 full knockout. Thus, it is possible that some alterations are not directly caused by lack of the Runt domain, but for instance, they may involve the remaining non-Runt domains of the protein which may still interact with regulatory proteins^65^. Indeed, the mutant Runx2 protein in 3G8 cells could still alter gene expression through indirect mechanisms, which are still relevant in understanding the role that Runx2 plays in melanoma. In conclusion, these findings support the idea that the Runt domain of Runx2 is involved in increasing the risk of tumour proliferation/migration and angiogenesis, and may be an ideal oncotarget in melanoma cancer cells.

## Supplementary information

Supplementary Info (Table 1, Fig 1)

Supplemental_Table_2 list of peptides area and of identified peptides

Supplemental_Table_3 list of normalized protein area and of identified proteins.xlsx

Supplemental_Table_4 List of differentially expressed proteins.xls

Supplemental_Table_5 GO enrichment analysis.xls

Supplemental_Table_6 IPA analysis.xls

## Data Availability

Data are available via ProteomeXchange with identifier PXD012096.
