# Publisher Correction: Runx2 stimulates neoangiogenesis through the Runt domain in melanoma

**DOI:** 10.1038/s41598-020-59556-5

**Published:** 2020-02-17

**Authors:** Daniela Cecconi, Jessica Brandi, Marcello Manfredi, Michela Serena, Luca Dalle Carbonare, Michela Deiana, Samuele Cheri, Francesca Parolini, Alberto Gandini, Giulia Marchetto, Giulio Innamorati, Francesco Avanzi, Franco Antoniazzi, Emilio Marengo, Natascia Tiso, Monica Mottes, Donato Zipeto, Maria Teresa Valenti

**Affiliations:** 1 Department of Biotechnology, Mass Spectrometry & Proteomics Lab, University of Verona, Verona, Italy; 2 Department of Sciences and Technological Innovation, University of Piemonte Orientale, Vercelli, Italy; 3 Department of Medicine, University of Verona, Verona, Italy; 4 Department of Neurosciences, Biomedicine and Movement Sciences, University of Verona, Verona, Italy; 5 Department of Surgery, Dentistry, Pediatrics and Gynecology, University of Verona, Verona, Italy; 6 Department of Biology, University of Padova, Padova, Italy

Correction to: *Scientific Reports* 10.1038/s41598-019-44552-1, published online 29 May 2019

In the original version of this Article, the author Luca Dalle Carbonare was incorrectly indexed. This error has now been corrected.

