# Supplementary material for: Runx2 stimulates neoangiogenesis through the Runt domain in melanoma
Source: Sci Rep. 2019 May 29;9:8052. doi: 10.1038/s41598-019-44552-1 (PMC6541657; doi:10.1038/s41598-019-44552-1)
Supplement: Supplementary file 1 — Supplementary Info (Table 1, Fig 1) [file 41598_2019_44552_MOESM1_ESM.pdf]

Runx2 stimulates neoangiogenesis through the RUNT domain in melanoma

Daniela Cecconi, Jessica Brandi, Marcello Manfredi, Michela Serena, Luca Dalle Carbonare, Michela Deiana, Samuele Cheri, Francesca Parolini, Alberto Gandini, Giulia Marchetto, Giulio Innamorati, Francesco Avanzi, Franco Antoniazzi, Emilio Marengo, Natascia Tiso, Monica Mottes, Donato Zipeto, Maria Teresa Valenti

Supplemental Table 1. List of antibodies used for western blotting validation

Antibody	Target	Western Blot	Origin	Secondary Antibody
**ANXA6**	Annexin VI	1:300	Santa Cruz (sc-271859)	Santa Cruz Anti-Mouse (sc-516102)
**ARHGEF1**	Rho Guanine Nucleotide exchange factor	1:300	Santa Cruz (sc-74565)	Santa Cruz Anti-Mouse (sc-516102)
**BID**	BH3 interactin domain-death agonist	1:300	Santa Cruz (sc-373939)	Santa Cruz Anti-Mouse (sc-516102)
**CAV1**	Caveolin 1	1:200	Santa Cruz (sc-53564)	Santa Cruz Anti-Mouse (sc-516102)
**FASN**	Fatty acid synthase	1:500	Santa Cruz (sc-55580)	Santa Cruz Anti-Mouse (sc-516102)
**IQGAP**	Ras GTPase-activating-like protein	1:300	Santa Cruz (sc-376021)	Santa Cruz Anti-Mouse (sc-516102)
**NSDHL**	NAD(P) Dependendent Steroid Deydrogenase-like	1:200	Santa Cruz (sc-390871)	Santa Cruz Anti-Mouse (sc-516102)
**PLIN2**	Perilipin-2	1:100	Santa Cruz (sc-377429)	Santa Cruz Anti-Mouse (sc-516102)
**RUNX2**	Runt related transcription factor 2	1:1000	Cell Signaling Technology (#8486)	Cell Signaling Technology Anti-Rabbit (#7074)
**SOAT1**	Sterol O-Acyltransferase	1:200	Santa Cruz (sc-137013)	Santa Cruz Anti-Mouse (sc-516102)

Supplementary Figure 1. Sequence of clones obtained by applying the CRISPR/Cas9 procedure to A375 cells.

A375 (WT) genomic sequence

GAGGGCATCTGGAAGCTGAATGAGGAAGACATGGAAATAATCTGAATATAGAGTCCTGGAAGTTCGTTGAGAGAAGGGATTGTT
ACCAAGGGGCAGGATCTTGGCAATGGATTAGGAGGAGTGTTTCATATATGACAGTGAAGGATCTGCACCAGCATCTCCTTACTGG
GCTCTGTGTTAATCAAAGGCACACATTTGCATGTTAGCTGACTCAGGTTAAAGTGTTATCTCTAGTCTTAGAGCAGAGAAAATGTTT
GTTTGTTCCAAAGATGGATTAATCTAGATCTTTTCTTGATTGAGTTAATTTGCTTCAGATTAACTGTTGAAGATTAAGCAGTCAAGTA
TGTCATTCCAGGATGGCAGATGGGACACAAGACATAATAGAACTGAGGCTAAGGGTATATATTGTTTTTTTCTTATTTATCATCAAT
TATTTTCACCTTCAGTTTCTTGATGACTTTGGATATCACACTGGGTCCCACCGCAGTGTGGTTTTCAGTAAATATACTTTATTAAAAG
CTGAAGTGCTAGCCCAAAGTAAGGAGATAAAACAGACTACACGATATCTAGGCTCTATGTAGTGAGGAGCCCCAGGGTTTCACTT
AAAAATCTTCTCCCATAAAGTTGACCAGACTTGATATTTTTTAGCCTGTGATGGGATAAGACCACACCTCAGGGCTCTCCCTGAGAG
AAAGCCTGCAGACAGGTGGTGCCTTGATCTGTATGATGCAATCTGCAGATGGGGCATCCCTCTTCCTTCTGTGGCATAATTCCTAC
GGACAGCTTAACATGGGGATTTAATAAAAATGTACATATTTTCTGTGAAGAATCCAACAACAAATGAAAGAAGTTTTTGCCTGCCT
TTGAAATGGGTGGACCCTGAAACATTGGTCTGTTTGAAGTGGCATCACAACCCATACACAGATGCTTCATTCCTGTCGGCCATTACT
GGACTGGACTAGAACACTAAGTCCTGATAAGACACATCATTTGCAATGAGAATATATTCTGTTTGTAAGCCTTTCTGATGTGCCATT
ATTGCTGCTGTGTTTCCTGTTTTATGTAGGTGGTAGCCCTCGGAGAGGTACCAGATGGGACTGTGGTTACTGTCATGGCGGGTAAC
GATGAAAATTATTCTGCTGAGCTCCGGAATGCCTCTGCTGTTATGAAAAACCAAGTAGCAAGGTTCAACGATCTGAGATTTGTGGG
CCGGAGTGGACGAGGTAGGTCTCTGACTTTTGATACTGATAATAGAATAAGCACATTAGGCTCCTTTGATGAAATGTAGACTAGTC
TGTATACAAATCAGCACCTTCTTTTTCTGAATAGAATTACTGAAGATTTGATTTAAATACATCCAGATGAAGTTGAGTGTTTTTCTGA
GTACTCAGGCCTTTTTCATTTATTTTATGATATTGAAAATTCAAACAATGTTTTCAGAAAAACTAGCTCCTAATTTCACTGGGTGTGA
GCATATAAGGTAGAGAAAAGAACAGTTGGAGGATCAACTTCTGAATTATGAGGAGTCAAATAACTGAGTAAATTGAGTGGTAAA
GGGGAAAGAAAAATGGATATGTTGGATCAATTTCCTGGTAATATCAATTTTGGTCTAAGTTGTAGCAAGGACAGCTGGCATAATG
CTCTTCACTTATCACAGGTTACCTCTATATGATTTAATTTACTCTCATAGTAAGAATTTATTAAAAACCATGGTTTTTTTTTAACATAA
GGCTTTTCATTTGAATAAGTGAATAATGTATTACTTATGTTAGAGTGACTATGTAAGGTTGCCTAGACAGATGAATGATTAGTACTC
TAATATTAATAGTTCCATACTATGTAAGGATTCCCATCATCTGAATCCATATTCAAAAATTAGAGATCCTTAAATAGAAAATTATCCC
TTTTAGGGTTTTTGTAACTGTTTGATAAATGAGGTTAATGTTTTTTAAAAAAGTCTTTAAGGAGGCCAATATTTTGGCTTTTATTTAC
TTATTCTACAGAAAGACTTTTACTAGTTACTCACTATTAGAAGCATTATGCTTATTTATTTACTTAACTGAATCTAATATTTCCATTTTA
AGTATTTTGATAAGAAGAAAGAAAATAGACCACTTTAGAACTGAGGTGAAATAAAGCCTTATGGAATCATTGCAACCCAGGTTTG
AATAGGAAATGAGGAGCACCACTTTTTAAACCATGTCTAATAATTGCTCTTTAGTTTGGATCAGATGTCAAATTATTACCATTGTAA
AAACTTGATAAGGGAAATTCAAGCAAGGCTGTTTCCGATGAGTAGTGTTAGTTTACTTTCTAGGAACAGGGCCAAGAAGTTGTACT
TTTTAACTTAAAAAAAATGATTCATGCTAATGAGATACTG

1F3 clone genomic sequence (as WT)

GAGGGCATCTGGAAGCTGAATGAGGAAGACATGGAAATAATCTGAATATAGAGTCCTGGAAGTTCGTTGAGAGAAGGGATTGTT
ACCAAGGGGCAGGATCTTGGCAATGGATTAGGAGGAGTGTTTCATATATGACAGTGAAGGATCTGCACCAGCATCTCCTTACTGG
GCTCTGTGTTAATCAAAGGCACACATTTGCATGTTAGCTGACTCAGGTTAAAGTGTTATCTCTAGTCTTAGAGCAGAGAAAATGTTT
GTTTGTTCCAAAGATGGATTAATCTAGATCTTTTCTTGATTGAGTTAATTTGCTTCAGATTAACTGTTGAAGATTAAGCAGTCAAGTA
TGTCATTCCAGGATGGCAGATGGGACACAAGACATAATAGAACTGAGGCTAAGGGTATATATTGTTTTTTTCTTATTTATCATCAAT
TATTTTCACCTTCAGTTTCTTGATGACTTTGGATATCACACTGGGTCCCACCGCAGTGTGGTTTTCAGTAAATATACTTTATTAAAAG
CTGAAGTGCTAGCCCAAAGTAAGGAGATAAAACAGACTACACGATATCTAGGCTCTATGTAGTGAGGAGCCCCAGGGTTTCACTT
AAAAATCTTCTCCCATAAAGTTGACCAGACTTGATATTTTTTAGCCTGTGATGGGATAAGACCACACCTCAGGGCTCTCCCTGAGAG
AAAGCCTGCAGACAGGTGGTGCCTTGATCTGTATGATGCAATCTGCAGATGGGGCATCCCTCTTCCTTCTGTGGCATAATTCCTAC
GGACAGCTTAACATGGGGATTTAATAAAAATGTACATATTTTCTGTGAAGAATCCAACAACAAATGAAAGAAGTTTTTGCCTGCCT
TTGAAATGGGTGGACCCTGAAACATTGGTCTGTTTGAAGTGGCATCACAACCCATACACAGATGCTTCATTCCTGTCGGCCATTACT
GGACTGGACTAGAACACTAAGTCCTGATAAGACACATCATTTGCAATGAGAATATATTCTGTTTGTAAGCCTTTCTGATGTGCCATT
ATTGCTGCTGTGTTTCCTGTTTTATGTAGGTGGTAGCCCTCGGAGAGGTACCAGATGGGACTGTGGTTACTGTCATGGCGGGTAAC
GATGAAAATTATTCTGCTGAGCTCCGGAATGCCTCTGCTGTTATGAAAAACCAAGTAGCAAGGTTCAACGATCTGAGATTTGTGGG
CCGGAGTGGACGAGGTAGGTCTCTGACTTTTGATACTGATAATAGAATAAGCACATTAGGCTCCTTTGATGAAATGTAGACTAGTC
TGTATACAAATCAGCACCTTCTTTTTCTGAATAGAATTACTGAAGATTTGATTTAAATACATCCAGATGAAGTTGAGTGTTTTTCTGA

GTACTCAGGCCTTTTTCATTTATTTTATGATATTGAAAATTCAAACAATGTTTTCAGAAAAACTAGCTCCTAATTTCACTGGGTGTGA
GCATATAAGGTAGAGAAAAGAACAGTTGGAGGATCAACTTCTGAATTATGAGGAGTCAAATAACTGAGTAAATTGAGTGGTAAA
GGGGAAAGAAAAATGGATATGTTGGATCAATTTCCTGGTAATATCAATTTTGGTCTAAGTTGTAGCAAGGACAGCTGGCATAATG
CTCTTCACTTATCACAGGTTACCTCTATATGATTTAATTTACTCTCATAGTAAGAATTTATTAAAAACCATGGTTTTTTTTTAACATAA
GGCTTTTCATTTGAATAAGTGAATAATGTATTACTTATGTTAGAGTGACTATGTAAGGTTGCCTAGACAGATGAATGATTAGTACTC
TAATATTAATAGTTCCATACTATGTAAGGATTCCCATCATCTGAATCCATATTCAAAAATTAGAGATCCTTAAATAGAAAATTATCCC
TTTTAGGGTTTTTGTAACTGTTTGATAAATGAGGTTAATGTTTTTTAAAAAAGTCTTTAAGGAGGCCAATATTTTGGCTTTTATTTAC
TTATTCTACAGAAAGACTTTTACTAGTTACTCACTATTAGAAGCATTATGCTTATTTATTTACTTAACTGAATCTAATATTTCCATTTTA
AGTATTTTGATAAGAAGAAAGAAAATAGACCACTTTAGAACTGAGGTGAAATAAAGCCTTATGGAATCATTGCAACCCAGGTTTG
AATAGGAAATGAGGAGCACCACTTTTTAAACCATGTCTAATAATTGCTCTTTAGTTTGGATCAGATGTCAAATTATTACCATTGTAA
AAACTTGATAAGGGAAATTCAAGCAAGGCTGTTTCCGATGAGTAGTGTTAGTTTACTTTCTAGGAACAGGGCCAAGAAGTTGTACT
TTTTAACTTAAAAAAAAATGATTCATGCTAATGAGATACTG

2D2 clone genomic sequence (as wt)

GAGGGCATCTGGAAGCTGAATGAGGAAGACATGGAAATAATCTGAATATAGAGTCCTGGAAGTTCGTTGAGAGAAGGGATTGTT
ACCAAGGGGCAGGATCTTGGCAATGGATTAGGAGGAGTGTTTCATATATGACAGTGAAGGATCTGCACCAGCATCTCCTTACTGG
GCTCTGTGTTAATCAAAGGCACACATTTGCATGTTAGCTGACTCAGGTTAAAGTGTTATCTCTAGTCTTAGAGCAGAGAAAATGTTT
GTTTGTTCCAAAGATGGATTAATCTAGATCTTTTCTTGATTGAGTTAATTTGCTTCAGATTAACTGTTGAAGATTAAGCAGTCAAGTA
TGTCATTCCAGGATGGCAGATGGGACACAAGACATAATAGAACTGAGGCTAAGGGTATATATTGTTTTTTTCTTATTTATCATCAAT
TATTTTCACCTTCAGTTTCTTGATGACTTTGGATATCACACTGGGTCCCACCGCAGTGTGGTTTTCAGTAAATATACTTTATTAAAAG
CTGAAGTGCTAGCCCAAAGTAAGGAGATAAAACAGACTACACGATATCTAGGCTCTATGTAGTGAGGAGCCCCAGGGTTTCACTT
AAAAATCTTCTCCCATAAAGTTGACCAGACTTGATATTTTTTAGCCTGTGATGGGATAAGACCACACCTCAGGGCTCTCCCTGAGAG
AAAGCCTGCAGACAGGTGGTGCCTTGATCTGTATGATGCAATCTGCAGATGGGGCATCCCTCTTCCTTCTGTGGCATAATTCCTAC
GGACAGCTTAACATGGGGATTTAATAAAAATGTACATATTTTCTGTGAAGAATCCAACAACAAATGAAAGAAGTTTTTGCCTGCCT
TTGAAATGGGTGGACCCTGAAACATTGGTCTGTTTGAAGTGGCATCACAACCCATACACAGATGCTTCATTCCTGTCGGCCATTACT
GGACTGGACTAGAACACTAAGTCCTGATAAGACACATCATTTGCAATGAGAATATATTCTGTTTGTAAGCCTTTCTGATGTGCCATT
ATTGCTGCTGTGTTTCCTGTTTTATGTAGGTGGTAGCCCTCGGAGAGGTACCAGATGGGACTGTGGTTACTGTCATGGCGGGTAAC
GATGAAAATTATTCTGCTGAGCTCCGGAATGCCTCTGCTGTTATGAAAAACCAAGTAGCAAGGTTCAACGATCTGAGATTTGTGGG
CCGGAGTGGACGAGGTAGGTCTCTGACTTTTGATACTGATAATAGAATAAGCACATTAGGCTCCTTTGATGAAATGTAGACTAGTC
TGTATACAAATCAGCACCTTCTTTTTCTGAATAGAATTACTGAAGATTTGATTTAAATACATCCAGATGAAGTTGAGTGTTTTTCTGA
GTACTCAGGCCTTTTTCATTTATTTTATGATATTGAAAATTCAAACAATGTTTTCAGAAAAACTAGCTCCTAATTTCACTGGGTGTGA
GCATATAAGGTAGAGAAAAGAACAGTTGGAGGATCAACTTCTGAATTATGAGGAGTCAAATAACTGAGTAAATTGAGTGGTAAA
GGGGAAAGAAAAATGGATATGTTGGATCAATTTCCTGGTAATATCAATTTTGGTCTAAGTTGTAGCAAGGACAGCTGGCATAATG
CTCTTCACTTATCACAGGTTACCTCTATATGATTTAATTTACTCTCATAGTAAGAATTTATTAAAAACCATGGTTTTTTTTTAACATAA
GGCTTTTCATTTGAATAAGTGAATAATGTATTACTTATGTTAGAGTGACTATGTAAGGTTGCCTAGACAGATGAATGATTAGTACTC
TAATATTAATAGTTCCATACTATGTAAGGATTCCCATCATCTGAATCCATATTCAAAAATTAGAGATCCTTAAATAGAAAATTATCCC
TTTTAGGGTTTTTGTAACTGTTTGATAAATGAGGTTAATGTTTTTTAAAAAAGTCTTTAAGGAGGCCAATATTTTGGCTTTTATTTAC
TTATTCTACAGAAAGACTTTTACTAGTTACTCACTATTAGAAGCATTATGCTTATTTATTTACTTAACTGAATCTAATATTTCCATTTTA
AGTATTTTGATAAGAAGAAAGAAAATAGACCACTTTAGAACTGAGGTGAAATAAAGCCTTATGGAATCATTGCAACCCAGGTTTG
AATAGGAAATGAGGAGCACCACTTTTTAAACCATGTCTAATAATTGCTCTTTAGTTTGGATCAGATGTCAAATTATTACCATTGTAA
AAACTTGATAAGGGAAATTCAAGCAAGGCTGTTTCCGATGAGTAGTGTTAGTTTACTTTCTAGGAACAGGGCCAAGAAGTTGTACT
TTTTAACTTAAAAAAAAATGATTCATGCTAATGAGATACTG

2E2 clone genomic sequence (as wt)

GAGGGCATCTGGAAGCTGAATGAGGAAGACATGGAAATAATCTGAATATAGAGTCCTGGAAGTTCGTTGAGAGAAGGGATTGTT
ACCAAGGGGCAGGATCTTGGCAATGGATTAGGAGGAGTGTTTCATATATGACAGTGAAGGATCTGCACCAGCATCTCCTTACTGG
GCTCTGTGTTAATCAAAGGCACACATTTGCATGTTAGCTGACTCAGGTTAAAGTGTTATCTCTAGTCTTAGAGCAGAGAAAATGTTT
GTTTGTTCCAAAGATGGATTAATCTAGATCTTTTCTTGATTGAGTTAATTTGCTTCAGATTAACTGTTGAAGATTAAGCAGTCAAGTA
TGTCATTCCAGGATGGCAGATGGGACACAAGACATAATAGAACTGAGGCTAAGGGTATATATTGTTTTTTTCTTATTTATCATCAAT

TATTTTCACCTTCAGTTTCTTGATGACTTTGGATATCACACTGGGTCCCACCGCAGTGTGGTTTTCAGTAAATATACTTTATTAAAAG
CTGAAGTGCTAGCCCAAAGTAAGGAGATAAAACAGACTACACGATATCTAGGCTCTATGTAGTGAGGAGCCCCAGGGTTTCACTT
AAAAATCTTCTCCCATAAAGTTGACCAGACTTGATATTTTTTAGCCTGTGATGGGATAAGACCACACCTCAGGGCTCTCCCTGAGAG
AAAGCCTGCAGACAGGTGGTGCCTTGATCTGTATGATGCAATCTGCAGATGGGGCATCCCTCTTCCTTCTGTGGCATAATTCCTAC
GGACAGCTTAACATGGGGATTTAATAAAAATGTACATATTTTCTGTGAAGAATCCAACAACAAATGAAAGAAGTTTTTGCCTGCCT
TTGAAATGGGTGGACCCTGAAACATTGGTCTGTTTGAAGTGGCATCACAACCCATACACAGATGCTTCATTCCTGTCGGCCATTACT
GGACTGGACTAGAACACTAAGTCCTGATAAGACACATCATTTGCAATGAGAATATATTCTGTTTGTAAGCCTTTCTGATGTGCCATT
ATTGCTGCTGTGTTTCCTGTTTTATGTAGGTGGTAGCCCTCGGAGAGGTACCAGATGGGACTGTGGTTACTGTCATGGCGGGTAAC
GATGAAAATTATTCTGCTGAGCTCCGGAATGCCTCTGCTGTTATGAAAAACCAAGTAGCAAGGTTCAACGATCTGAGATTTGTGGG
CCGGAGTGGACGAGGTAGGTCTCTGACTTTTGATACTGATAATAGAATAAGCACATTAGGCTCCTTTGATGAAATGTAGACTAGTC
TGTATACAAATCAGCACCTTCTTTTTCTGAATAGAATTACTGAAGATTTGATTTAAATACATCCAGATGAAGTTGAGTGTTTTTCTGA
GTACTCAGGCCTTTTTCATTTATTTTATGATATTGAAAATTCAAACAATGTTTTCAGAAAAACTAGCTCCTAATTTCACTGGGTGTGA
GCATATAAGGTAGAGAAAAGAACAGTTGGAGGATCAACTTCTGAATTATGAGGAGTCAAATAACTGAGTAAATTGAGTGGTAAA
GGGGAAAGAAAAATGGATATGTTGGATCAATTTCCTGGTAATATCAATTTTGGTCTAAGTTGTAGCAAGGACAGCTGGCATAATG
CTCTTCACTTATCACAGGTTACCTCTATATGATTTAATTTACTCTCATAGTAAGAATTTATTAAAAACCATGGTTTTTTTTAACATAA
GGCTTTTCATTTGAATAAGTGAATAATGTATTACTTATGTTAGAGTGACTATGTAAGGTTGCCTAGACAGATGAATGATTAGTACTC
TAATATTAATAGTTCCATACTATGTAAGGATTCCCATCATCTGAATCCATATTCAAAAATTAGAGATCCTTAAATAGAAAATTATCCC
TTTTAGGGTTTTTGTAACTGTTTGATAAATGAGGTTAATGTTTTTTAAAAAAGTCTTTAAGGAGGCCAATATTTTGGCTTTTATTTAC
TTATTCTACAGAAAGACTTTTACTAGTTACTCACTATTAGAAGCATTATGCTTATTTATTTACTTAACTGAATCTAATATTTCCATTTTA
AGTATTTTGATAAGAAGAAAGAAAATAGACCACTTTAGAACTGAGGTGAAATAAAGCCTTATGGAATCATTGCAACCCAGGTTTG
AATAGGAAATGAGGAGCACCACTTTTTAAACCATGTCTAATAATTGCTCTTTAGTTTGGATCAGATGTCAAATTATTACCATTGTAA
AAACTTGATAAGGGAAATTCAAGCAAGGCTGTTTCCGATGAGTAGTGTTAGTTTACTTTCTAGGAACAGGGCCAAGAAGTTGTACT
TTTTAACTTAAAAAAAATGATTCATGCTAATGAGATACTG

2E2(n) clone genomic sequence (as wt)

GAGGGCATCTGGAAGCTGAATGAGGAAGACATGGAAATAATCTGAATATAGAGTCCTGGAAGTTCGTTGAGAGAAGGGATTGTT
ACCAAGGGGCAGGATCTTGGCAATGGATTAGGAGGAGTGTTTCATATATGACAGTGAAGGATCTGCACCAGCATCTCCTTACTGG
GCTCTGTGTTAATCAAAGGCACACATTTGCATGTTAGCTGACTCAGGTTAAAGTGTTATCTCTAGTCTTAGAGCAGAGAAAATGTTT
GTTTGTTCCAAAGATGGATTAATCTAGATCTTTTCTTGATTGAGTTAATTTGCTTCAGATTAACTGTTGAAGATTAAGCAGTCAAGTA
TGTCATTCCAGGATGGCAGATGGGACACAAGACATAATAGAACTGAGGCTAAGGGTATATATTGTTTTTTTCTTATTTATCATCAAT
TATTTTCACCTTCAGTTTCTTGATGACTTTGGATATCACACTGGGTCCCACCGCAGTGTGGTTTTCAGTAAATATACTTTATTAAAAG
CTGAAGTGCTAGCCCAAAGTAAGGAGATAAAACAGACTACACGATATCTAGGCTCTATGTAGTGAGGAGCCCCAGGGTTTCACTT
AAAAATCTTCTCCCATAAAGTTGACCAGACTTGATATTTTTTAGCCTGTGATGGGATAAGACCACACCTCAGGGCTCTCCCTGAGAG
AAAGCCTGCAGACAGGTGGTGCCTTGATCTGTATGATGCAATCTGCAGATGGGGCATCCCTCTTCCTTCTGTGGCATAATTCCTAC
GGACAGCTTAACATGGGGATTTAATAAAAATGTACATATTTTCTGTGAAGAATCCAACAACAAATGAAAGAAGTTTTTGCCTGCCT
TTGAAATGGGTGGACCCTGAAACATTGGTCTGTTTGAAGTGGCATCACAACCCATACACAGATGCTTCATTCCTGTCGGCCATTACT
GGACTGGACTAGAACACTAAGTCCTGATAAGACACATCATTTGCAATGAGAATATATTCTGTTTGTAAGCCTTTCTGATGTGCCATT
ATTGCTGCTGTGTTTCCTGTTTTATGTAGGTGGTAGCCCTCGGAGAGGTACCAGATGGGACTGTGGTTACTGTCATGGCGGGTAAC
GATGAAAATTATTCTGCTGAGCTCCGGAATGCCTCTGCTGTTATGAAAAACCAAGTAGCAAGGTTCAACGATCTGAGATTTGTGGG
CCGGAGTGGACGAGGTAGGTCTCTGACTTTTGATACTGATAATAGAATAAGCACATTAGGCTCCTTTGATGAAATGTAGACTAGTC
TGTATACAAATCAGCACCTTCTTTTTCTGAATAGAATTACTGAAGATTTGATTTAAATACATCCAGATGAAGTTGAGTGTTTTTCTGA
GTACTCAGGCCTTTTTCATTTATTTTATGATATTGAAAATTCAAACAATGTTTTCAGAAAAACTAGCTCCTAATTTCACTGGGTGTGA
GCATATAAGGTAGAGAAAAGAACAGTTGGAGGATCAACTTCTGAATTATGAGGAGTCAAATAACTGAGTAAATTGAGTGGTAAA
GGGGAAAGAAAAATGGATATGTTGGATCAATTTCCTGGTAATATCAATTTTGGTCTAAGTTGTAGCAAGGACAGCTGGCATAATG
CTCTTCACTTATCACAGGTTACCTCTATATGATTTAATTTACTCTCATAGTAAGAATTTATTAAAAACCATGGTTTTTTTTAACATAA
GGCTTTTCATTTGAATAAGTGAATAATGTATTACTTATGTTAGAGTGACTATGTAAGGTTGCCTAGACAGATGAATGATTAGTACTC
TAATATTAATAGTTCCATACTATGTAAGGATTCCCATCATCTGAATCCATATTCAAAAATTAGAGATCCTTAAATAGAAAATTATCCC
TTTTAGGGTTTTTGTAACTGTTTGATAAATGAGGTTAATGTTTTTTAAAAAAGTCTTTAAGGAGGCCAATATTTTGGCTTTTATTTAC
TTATTCTACAGAAAGACTTTTACTAGTTACTCACTATTAGAAGCATTATGCTTATTTATTTACTTAACTGAATCTAATATTTCCATTTTA
AGTATTTTGATAAGAAGAAAGAAAATAGACCACTTTAGAACTGAGGTGAAATAAAGCCTTATGGAATCATTGCAACCCAGGTTTG
AATAGGAAATGAGGAGCACCACTTTTTAAACCATGTCTAATAATTGCTCTTTAGTTTGGATCAGATGTCAAATTATTACCATTGTAA
AAACTTGATAAGGGAAATTCAAGCAAGGCTGTTTCCGATGAGTAGTGTTAGTTTACTTTCTAGGAACAGGGCCAAGAAGTTGTACT
TTTTAACTTAAAAAAAATGATTCATGCTAATGAGATACTG

3G4 clone genomic sequence (as wt)

GAGGGCATCTGGAAGCTGAATGAGGAAGACATGGAAATAATCTGAATATAGAGTCCTGGAAGTTCGTTGAGAGAAGGGATTGTT
ACCAAGGGGCAGGATCTTGGCAATGGATTAGGAGGAGTGTTTCATATATGACAGTGAAGGATCTGCACCAGCATCTCCTTACTGG
GCTCTGTGTTAATCAAAGGCACACATTTGCATGTTAGCTGACTCAGGTTAAAGTGTTATCTCTAGTCTTAGAGCAGAGAAAATGTTT
GTTTGTTCCAAAGATGGATTAATCTAGATCTTTTCTTGATTGAGTTAATTTGCTTCAGATTAACTGTTGAAGATTAAGCAGTCAAGTA
TGTCATTCCAGGATGGCAGATGGGACACAAGACATAATAGAACTGAGGCTAAGGGTATATATTGTTTTTTTCTTATTTATCATCAAT
TATTTTCACCTTCAGTTTCTTGATGACTTTGGATATCACACTGGGTCCCACCGCAGTGTGGTTTTCAGTAAATATACTTTATTAAAAG
CTGAAGTGCTAGCCCAAAGTAAGGAGATAAAACAGACTACACGATATCTAGGCTCTATGTAGTGAGGAGCCCCAGGGTTTCACTT
AAAAATCTTCTCCCATAAAGTTGACCAGACTTGATATTTTTTAGCCTGTGATGGGATAAGACCACACCTCAGGGCTCTCCCTGAGAG
AAAGCCTGCAGACAGGTGGTGCCTTGATCTGTATGATGCAATCTGCAGATGGGGCATCCCTCTTCCTTCTGTGGCATAATTCCTAC
GGACAGCTTAACATGGGGATTTAATAAAAATGTACATATTTTCTGTGAAGAATCCAACAACAAATGAAAGAAGTTTTTGCCTGCCT
TTGAAATGGGTGGACCCTGAAACATTGGTCTGTTTGAAGTGGCATCACAACCCATACACAGATGCTTCATTCCTGTCGGCCATTACT
GGACTGGACTAGAACACTAAGTCCTGATAAGACACATCATTTGCAATGAGAATATATTCTGTTTGTAAGCCTTTCTGATGTGCCATT
ATTGCTGCTGTGTTTCCTGTTTTATGTAGGTGGTAGCCCTCGGAGAGGTACCAGATGGGACTGTGGTTACTGTCATGGCGGGTAAC
GATGAAAATTATTCTGCTGAGCTCCGGAATGCCTCTGCTGTTATGAAAAACCAAGTAGCAAGGTTCAACGATCTGAGATTTGTGGG
CCGGAGTGGACGAGGTAGGTCTCTGACTTTTGATACTGATAATAGAATAAGCACATTAGGCTCCTTTGATGAAATGTAGACTAGTC
TGTATACAAATCAGCACCTTCTTTTTCTGAATAGAATTACTGAAGATTTGATTTAAATACATCCAGATGAAGTTGAGTGTTTTTCTGA
GTACTCAGGCCTTTTTCATTTATTTTATGATATTGAAAATTCAAACAATGTTTTCAGAAAAACTAGCTCCTAATTTCACTGGGTGTGA
GCATATAAGGTAGAGAAAAGAACAGTTGGAGGATCAACTTCTGAATTATGAGGAGTCAAATAACTGAGTAAATTGAGTGGTAAA
GGGGAAAGAAAAATGGATATGTTGGATCAATTTCCTGGTAATATCAATTTTGGTCTAAGTTGTAGCAAGGACAGCTGGCATAATG
CTCTTCACTTATCACAGGTTACCTCTATATGATTTAATTTACTCTCATAGTAAGAATTTATTAAAAACCATGGTTTTTTTTTAACATAA
GGCTTTTCATTTGAATAAGTGAATAATGTATTACTTATGTTAGAGTGACTATGTAAGGTTGCCTAGACAGATGAATGATTAGTACTC
TAATATTAATAGTTCCATACTATGTAAGGATTCCCATCATCTGAATCCATATTCAAAAATTAGAGATCCTTAAATAGAAAATTATCCC
TTTTAGGGTTTTTGTAACTGTTTGATAAATGAGGTTAATGTTTTTTAAAAAAGTCTTTAAGGAGGCCAATATTTTGGCTTTTATTTAC
TTATTCTACAGAAAGACTTTTACTAGTTACTCACTATTAGAAGCATTATGCTTATTTATTTACTTAACTGAATCTAATATTTCCATTTTA
AGTATTTTGATAAGAAGAAAGAAAATAGACCACTTTAGAACTGAGGTGAAATAAAGCCTTATGGAATCATTGCAACCCAGGTTTG
AATAGGAAATGAGGAGCACCACTTTTTAAACCATGTCTAATAATTGCTCTTTAGTTTGGATCAGATGTCAAATTATTACCATTGTAA
AAACTTGATAAGGGAAATTCAAGCAAGGCTGTTTCCGATGAGTAGTGTTAGTTTACTTTCTAGGAACAGGGCCAAGAAGTTGTACT
TTTTAACTTAAAAAAAAATGATTCATGCTAATGAGATACTG

3G8 clone genomic sequence (deletion in red)

GAGGGCATCTGGAAGCTGAATGAGGAAGACATGGAAATAATCTGAATATAGAGTCCTGGAAGTTCGTTGAGAGAAGGGATTGTT
ACCAAGGGGCAGGATCTTGGCAATGGATTAGGAGGAGTGTTTCATATATGACAGTGAAGGATCTGCACCAGCATCTCCTTACTGG
GCTCTGTGTTAATCAAAGGCACACATTTGCATGTTAGCTGACTCAGGTTAAAGTGTTATCTCTAGTCTTAGAGCAGAGAAAATGTTT
GTTTGTTCCAAAGATGGATTAATCTAGATCTTTTCTTGATTGAGTTAATTTGCTTCAGATTAACTGTTGAAGATTAAGCAGTCAAGTA
TGTCATTCCAGGATGGCAGATGGGACACAAGACATAATAGAACTGAGGCTAAGGGTATATATTGTTTTTTTCTTATTTATCATCAAT
TATTTTCACCTTCAGTTTCTTGATGACTTTGGATATCACACTGGGTCCCACCGCAGTGTGGTTTTCAGTAAATATACTTTATTAAAAG
CTGAAGTGCTAGCCCAAAGTAAGGAGATAAAACAGACTACACGATATCTAGGCTCTATGTAGTGAGGAGCCCCAGGGTTTCACTT
AAAAATCTTCTCCCATAAAGTTGACCAGACTTGATATTTTTTAGCCTGTGATGGGATAAGACCACACCTCAGGGCTCTCCCTGAGAG
AAAGCCTGCAGACAGGTGGTGCCTTGATCTGTATGATGCAATCTGCAGATGGGGCATCCCTCTTCCTTCTGTGGCATAATTCCTAC
GGACAGCTTAACATGGGGATTTAATAAAAATGTACATATTTTCTGTGAAGAATCCAACAACAAATGAAAGAAGTTTTTGCCTGCCT
TTGAAATGGGTGGACCCTGAAACATTGGTCTGTTTGAAGTGGCATCACAACCCATACACAGATGCTTCATTCCTGTCGGCCATTACT
GGACTGGACTAGAACACTAAGTCCTGATAAGACACATCATTTGCAATGAGAATATATTCTGTTTGTAAGCCTTTCTGATGTGCCATT
ATTGCTGCTGTGTTTCCTGTTTTATGTAGG**TGGTAGCCCTCGGAGAGGTACCAGATGGGACTGTGGTTACTGTCATGGCGGGTAA**
CGATGAAAATTATTCTGCTGAGCTCCGGAATGCCTCTGCTGTTATGAAAAACCAAGTAGCAAGGTTCAACGATCTGAGATTTGTG
GGCCGGAGTGGACGAGGTAGGTCTCTGACTTTTGATACTGATAATAGAATAAGCACATTAGGCTCCTTTGATGAAATGTAGACTA
GTCTGTATACAAATCAGCACCTTCTTTTTCTGAATAGAATTACTGAAGATTTGATTTAAATACATCCAGATGAAGTTGAGTGTTTTTC
TGAGTACTCAGGCCTTTTTCATTTATTTTATGATATTGAAAATTCAAACAATGTTTTCAGAAAAACTAGCTCCTAATTTCACTGGGTG
TGAGCATATAAGGTAGAGAAAAGAACAGTTGGAGGATCAACTTCTGAATTATGAGGAGTCAAATAACTGAGTAAATTGAGTGGTA
AAGGGGAAAGAAAAATGGATATGTTGGATCAATTTCCTGGTAATATCAATTTTGGTCTAAGTTGTAGCAAGGACAGCTGGCATAA
TGCTCTTCACTTATCACAGGTTACCTCTATATGATTTAATTTACTCTCATAGTAAGAATTTATTAAAAACCATGGTTTTTTTTTAACAT
AAGGCTTTTCATTTGAATAAGTGAATAATGTATTACTTATGTTAGAGTGACTATGTAAGGTTGCCTAGACAGATGAATGATTAGTAC
TCTAATATTAATAGTTCCATACTATGTAAGGATTCCCATCATCTGAATCCATATTCAAAAATTAGAGATCCTTAAATAGAAAATTATC

CCTTTTAGGGTTTTTGTAACTGTTTGATAAATGAGGTTAATGTTTTTTAAAAAAGTCTTTAAGGAGGCCAATATTTTGGCTTTTATTT
ACTTATTCTACAGAAAGACTTTTACTAGTTACTCACTATTAGAAGCATTATGCTTATTTATTTACTTAACTGAATCTAATATTTCCATT
TTAAGTATTTTGATAAGAAGAAAGAAAATAGACCACTTTAGAACTGAGGTGAAATAAAGCCTTATGGAATCATTGCAACCCAGGTT
TGAATAGGAAATGAGGAGCACCACTTTTTAAACCATGTCTAATAATTGCTCTTTAGTTTGGATCAGATGTCAAATTATTACCATTGT
AAAAACTTGATAAGGGAAATTCAAGCAAGGCTGTTTCCGATGAGTAGTGTTAGTTTACTTTCTAGGAACAGGGCCAAGAAGTTGT
ACTTTTTAACTTAAAAAAAATGATTCATGCTAATGAGATACTG

4C7 clone genomic sequence (as wt)

GAGGGCATCTGGAAGCTGAATGAGGAAGACATGGAAATAATCTGAATATAGAGTCCTGGAAGTTCGTTGAGAGAAGGGATTGTT
ACCAAGGGGCAGGATCTTGGCAATGGATTAGGAGGAGTGTTTCATATATGACAGTGAAGGATCTGCACCAGCATCTCCTTACTGG
GCTCTGTGTTAATCAAAGGCACACATTTGCATGTTAGCTGACTCAGGTTAAAGTGTTATCTCTAGTCTTAGAGCAGAGAAAATGTTT
GTTTGTTCCAAAGATGGATTAATCTAGATCTTTTCTTGATTGAGTTAATTTGCTTCAGATTAACTGTTGAAGATTAAGCAGTCAAGTA
TGTCATTCCAGGATGGCAGATGGGACACAAGACATAATAGAACTGAGGCTAAGGGTATATATTGTTTTTTTCTTATTTATCATCAAT
TATTTTCACCTTCAGTTTCTTGATGACTTTGGATATCACACTGGGTCCCACCGCAGTGTGGTTTTCAGTAAATATACTTTATTAAAAG
CTGAAGTGCTAGCCCAAAGTAAGGAGATAAAACAGACTACACGATATCTAGGCTCTATGTAGTGAGGAGCCCCAGGGTTTCACTT
AAAAATCTTCTCCCATAAAGTTGACCAGACTTGATATTTTTTAGCCTGTGATGGGATAAGACCACACCTCAGGGCTCTCCCTGAGAG
AAAGCCTGCAGACAGGTGGTGCCTTGATCTGTATGATGCAATCTGCAGATGGGGCATCCCTCTTCCTTCTGTGGCATAATTCCTAC
GGACAGCTTAACATGGGGATTTAATAAAAATGTACATATTTTCTGTGAAGAATCCAACAACAAATGAAAGAAGTTTTTGCCTGCCT
TTGAAATGGGTGGACCCTGAAACATTGGTCTGTTTGAAGTGGCATCACAACCCATACACAGATGCTTCATTCCTGTCGGCCATTACT
GGACTGGACTAGAACACTAAGTCCTGATAAGACACATCATTTGCAATGAGAATATATTCTGTTTGTAAGCCTTTCTGATGTGCCATT
ATTGCTGCTGTGTTTCCTGTTTTATGTAGGTGGTAGCCCTCGGAGAGGTACCAGATGGGACTGTGGTTACTGTCATGGCGGGTAAC
GATGAAAATTATTCTGCTGAGCTCCGGAATGCCTCTGCTGTTATGAAAAACCAAGTAGCAAGGTTCAACGATCTGAGATTTGTGGG
CCGGAGTGGACGAGGTAGGTCTCTGACTTTTGATACTGATAATAGAATAAGCACATTAGGCTCCTTTGATGAAATGTAGACTAGTC
TGTATACAAATCAGCACCTTCTTTTTCTGAATAGAATTACTGAAGATTTGATTTAAATACATCCAGATGAAGTTGAGTGTTTTTCTGA
GTACTCAGGCCTTTTTCATTTATTTTATGATATTGAAAATTCAAACAATGTTTTCAGAAAAACTAGCTCCTAATTTCACTGGGTGTGA
GCATATAAGGTAGAGAAAAGAACAGTTGGAGGATCAACTTCTGAATTATGAGGAGTCAAATAACTGAGTAAATTGAGTGGTAAA
GGGGAAAGAAAAATGGATATGTTGGATCAATTTCCTGGTAATATCAATTTTGGTCTAAGTTGTAGCAAGGACAGCTGGCATAATG
CTCTTCACTTATCACAGGTTACCTCTATATGATTTAATTTACTCTCATAGTAAGAATTTATTAAAAACCATGGTTTTTTTTTAACATAA
GGCTTTTCATTTGAATAAGTGAATAATGTATTACTTATGTTAGAGTGACTATGTAAGGTTGCCTAGACAGATGAATGATTAGTACTC
TAATATTAATAGTTCCATACTATGTAAGGATTCCCATCATCTGAATCCATATTCAAAAATTAGAGATCCTTAAATAGAAAATTATCCC
TTTTAGGGTTTTTGTAACTGTTTGATAAATGAGGTTAATGTTTTTTAAAAAAGTCTTTAAGGAGGCCAATATTTTGGCTTTTATTTAC
TTATTCTACAGAAAGACTTTTACTAGTTACTCACTATTAGAAGCATTATGCTTATTTATTTACTTAACTGAATCTAATATTTCCATTTTA
AGTATTTTGATAAGAAGAAAGAAAATAGACCACTTTAGAACTGAGGTGAAATAAAGCCTTATGGAATCATTGCAACCCAGGTTTG
AATAGGAAATGAGGAGCACCACTTTTTAAACCATGTCTAATAATTGCTCTTTAGTTTGGATCAGATGTCAAATTATTACCATTGTAA
AAACTTGATAAGGGAAATTCAAGCAAGGCTGTTTCCGATGAGTAGTGTTAGTTTACTTTCTAGGAACAGGGCCAAGAAGTTGTACT
TTTTAACTTAAAAAAAATGATTCATGCTAATGAGATACTG

4D7 clone genomic sequence (as wt)

GAGGGCATCTGGAAGCTGAATGAGGAAGACATGGAAATAATCTGAATATAGAGTCCTGGAAGTTCGTTGAGAGAAGGGATTGTT
ACCAAGGGGCAGGATCTTGGCAATGGATTAGGAGGAGTGTTTCATATATGACAGTGAAGGATCTGCACCAGCATCTCCTTACTGG
GCTCTGTGTTAATCAAAGGCACACATTTGCATGTTAGCTGACTCAGGTTAAAGTGTTATCTCTAGTCTTAGAGCAGAGAAAATGTTT
GTTTGTTCCAAAGATGGATTAATCTAGATCTTTTCTTGATTGAGTTAATTTGCTTCAGATTAACTGTTGAAGATTAAGCAGTCAAGTA
TGTCATTCCAGGATGGCAGATGGGACACAAGACATAATAGAACTGAGGCTAAGGGTATATATTGTTTTTTTCTTATTTATCATCAAT
TATTTTCACCTTCAGTTTCTTGATGACTTTGGATATCACACTGGGTCCCACCGCAGTGTGGTTTTCAGTAAATATACTTTATTAAAAG
CTGAAGTGCTAGCCCAAAGTAAGGAGATAAAACAGACTACACGATATCTAGGCTCTATGTAGTGAGGAGCCCCAGGGTTTCACTT
AAAAATCTTCTCCCATAAAGTTGACCAGACTTGATATTTTTTAGCCTGTGATGGGATAAGACCACACCTCAGGGCTCTCCCTGAGAG
AAAGCCTGCAGACAGGTGGTGCCTTGATCTGTATGATGCAATCTGCAGATGGGGCATCCCTCTTCCTTCTGTGGCATAATTCCTAC
GGACAGCTTAACATGGGGATTTAATAAAAATGTACATATTTTCTGTGAAGAATCCAACAACAAATGAAAGAAGTTTTTGCCTGCCT
TTGAAATGGGTGGACCCTGAAACATTGGTCTGTTTGAAGTGGCATCACAACCCATACACAGATGCTTCATTCCTGTCGGCCATTACT
GGACTGGACTAGAACACTAAGTCCTGATAAGACACATCATTTGCAATGAGAATATATTCTGTTTGTAAGCCTTTCTGATGTGCCATT
ATTGCTGCTGTGTTTCCTGTTTTATGTAGGTGGTAGCCCTCGGAGAGGTACCAGATGGGACTGTGGTTACTGTCATGGCGGGTAAC
GATGAAAATTATTCTGCTGAGCTCCGGAATGCCTCTGCTGTTATGAAAAACCAAGTAGCAAGGTTCAACGATCTGAGATTTGTGGG
CCGGAGTGGACGAGGTAGGTCTCTGACTTTTGATACTGATAATAGAATAAGCACATTAGGCTCCTTTGATGAAATGTAGACTAGTC

TGTATACAAATCAGCACCTTCTTTTTCTGAATAGAATTACTGAAGATTTGATTTAAATACATCCAGATGAAGTTGAGTGTTTTTCTGA
GTACTCAGGCCTTTTTCATTTATTTTATGATATTGAAAATTCAAACAATGTTTTCAGAAAAACTAGCTCCTAATTTCACTGGGTGTGA
GCATATAAGGTAGAGAAAAGAACAGTTGGAGGATCAACTTCTGAATTATGAGGAGTCAAATAACTGAGTAAATTGAGTGGTAAA
GGGGAAAGAAAAATGGATATGTTGGATCAATTTCCTGGTAATATCAATTTTGGTCTAAGTTGTAGCAAGGACAGCTGGCATAATG
CTCTTCACTTATCACAGGTTACCTCTATATGATTTAATTTACTCTCATAGTAAGAATTTATTAAAAACCATGGTTTTTTTTTAACATAA
GGCTTTTCATTTGAATAAGTGAATAATGTATTACTTATGTTAGAGTGACTATGTAAGGTTGCCTAGACAGATGAATGATTAGTACTC
TAATATTAATAGTTCCATACTATGTAAGGATTCCCATCATCTGAATCCATATTCAAAAATTAGAGATCCTTAAATAGAAAATTATCCC
TTTTAGGGTTTTTGTAACTGTTTGATAAATGAGGTTAATGTTTTTTAAAAAAGTCTTTAAGGAGGCCAATATTTTGGCTTTTATTTAC
TTATTCTACAGAAAGACTTTTACTAGTTACTCACTATTAGAAGCATTATGCTTATTTATTTACTTAACTGAATCTAATATTTCCATTTTA
AGTATTTTGATAAGAAGAAAGAAAATAGACCACTTTAGAACTGAGGTGAAATAAAGCCTTATGGAATCATTGCAACCCAGGTTTG
AATAGGAAATGAGGAGCACCACTTTTTAAACCATGTCTAATAATTGCTCTTTAGTTTGGATCAGATGTCAAATTATTACCATTGTAA
AAACTTGATAAGGGAAATTCAAGCAAGGCTGTTTCCGATGAGTAGTGTTAGTTTACTTTCTAGGAACAGGGCCAAGAAGTTGTACT
TTTTAACTTAAAAAAAAATGATTCATGCTAATGAGATACTG

4E3 clone genomic sequence (as wt)

GAGGGCATCTGGAAGCTGAATGAGGAAGACATGGAAATAATCTGAATATAGAGTCCTGGAAGTTCGTTGAGAGAAGGGATTGTT
ACCAAGGGGCAGGATCTTGGCAATGGATTAGGAGGAGTGTTTCATATATGACAGTGAAGGATCTGCACCAGCATCTCCTTACTGG
GCTCTGTGTTAATCAAAGGCACACATTTGCATGTTAGCTGACTCAGGTTAAAGTGTTATCTCTAGTCTTAGAGCAGAGAAAATGTTT
GTTTGTTCCAAAGATGGATTAATCTAGATCTTTTCTTGATTGAGTTAATTTGCTTCAGATTAACTGTTGAAGATTAAGCAGTCAAGTA
TGTCATTCCAGGATGGCAGATGGGACACAAGACATAATAGAACTGAGGCTAAGGGTATATATTGTTTTTTTCTTATTTATCATCAAT
TATTTTCACCTTCAGTTTCTTGATGACTTTGGATATCACACTGGGTCCCACCGCAGTGTGGTTTTCAGTAAATATACTTTATTAAAAG
CTGAAGTGCTAGCCCAAAGTAAGGAGATAAAACAGACTACACGATATCTAGGCTCTATGTAGTGAGGAGCCCCAGGGTTTCACTT
AAAAATCTTCTCCCATAAAGTTGACCAGACTTGATATTTTTTAGCCTGTGATGGGATAAGACCACACCTCAGGGCTCTCCCTGAGAG
AAAGCCTGCAGACAGGTGGTGCCTTGATCTGTATGATGCAATCTGCAGATGGGGCATCCCTCTTCCTTCTGTGGCATAATTCCTAC
GGACAGCTTAACATGGGGATTTAATAAAAATGTACATATTTTCTGTGAAGAATCCAACAACAAATGAAAGAAGTTTTTGCCTGCCT
TTGAAATGGGTGGACCCTGAAACATTGGTCTGTTTGAAGTGGCATCACAACCCATACACAGATGCTTCATTCCTGTCGGCCATTACT
GGACTGGACTAGAACACTAAGTCCTGATAAGACACATCATTTGCAATGAGAATATATTCTGTTTGTAAGCCTTTCTGATGTGCCATT
ATTGCTGCTGTGTTTCCTGTTTTATGTAGGTGGTAGCCCTCGGAGAGGTACCAGATGGGACTGTGGTTACTGTCATGGCGGGTAAC
GATGAAAATTATTCTGCTGAGCTCCGGAATGCCTCTGCTGTTATGAAAAACCAAGTAGCAAGGTTCAACGATCTGAGATTTGTGGG
CCGGAGTGGACGAGGTAGGTCTCTGACTTTTGATACTGATAATAGAATAAGCACATTAGGCTCCTTTGATGAAATGTAGACTAGTC
TGTATACAAATCAGCACCTTCTTTTTCTGAATAGAATTACTGAAGATTTGATTTAAATACATCCAGATGAAGTTGAGTGTTTTTCTGA
GTACTCAGGCCTTTTTCATTTATTTTATGATATTGAAAATTCAAACAATGTTTTCAGAAAAACTAGCTCCTAATTTCACTGGGTGTGA
GCATATAAGGTAGAGAAAAGAACAGTTGGAGGATCAACTTCTGAATTATGAGGAGTCAAATAACTGAGTAAATTGAGTGGTAAA
GGGGAAAGAAAAATGGATATGTTGGATCAATTTCCTGGTAATATCAATTTTGGTCTAAGTTGTAGCAAGGACAGCTGGCATAATG
CTCTTCACTTATCACAGGTTACCTCTATATGATTTAATTTACTCTCATAGTAAGAATTTATTAAAAACCATGGTTTTTTTTTAACATAA
GGCTTTTCATTTGAATAAGTGAATAATGTATTACTTATGTTAGAGTGACTATGTAAGGTTGCCTAGACAGATGAATGATTAGTACTC
TAATATTAATAGTTCCATACTATGTAAGGATTCCCATCATCTGAATCCATATTCAAAAATTAGAGATCCTTAAATAGAAAATTATCCC
TTTTAGGGTTTTTGTAACTGTTTGATAAATGAGGTTAATGTTTTTTAAAAAAGTCTTTAAGGAGGCCAATATTTTGGCTTTTATTTAC
TTATTCTACAGAAAGACTTTTACTAGTTACTCACTATTAGAAGCATTATGCTTATTTATTTACTTAACTGAATCTAATATTTCCATTTTA
AGTATTTTGATAAGAAGAAAGAAAATAGACCACTTTAGAACTGAGGTGAAATAAAGCCTTATGGAATCATTGCAACCCAGGTTTG
AATAGGAAATGAGGAGCACCACTTTTTAAACCATGTCTAATAATTGCTCTTTAGTTTGGATCAGATGTCAAATTATTACCATTGTAA
AAACTTGATAAGGGAAATTCAAGCAAGGCTGTTTCCGATGAGTAGTGTTAGTTTACTTTCTAGGAACAGGGCCAAGAAGTTGTACT
TTTTAACTTAAAAAAAAATGATTCATGCTAATGAGATACTG

4E4 clone genomic sequence (as wt)

GAGGGCATCTGGAAGCTGAATGAGGAAGACATGGAAATAATCTGAATATAGAGTCCTGGAAGTTCGTTGAGAGAAGGGATTGTT
ACCAAGGGGCAGGATCTTGGCAATGGATTAGGAGGAGTGTTTCATATATGACAGTGAAGGATCTGCACCAGCATCTCCTTACTGG
GCTCTGTGTTAATCAAAGGCACACATTTGCATGTTAGCTGACTCAGGTTAAAGTGTTATCTCTAGTCTTAGAGCAGAGAAAATGTTT
GTTTGTTCCAAAGATGGATTAATCTAGATCTTTTCTTGATTGAGTTAATTTGCTTCAGATTAACTGTTGAAGATTAAGCAGTCAAGTA
TGTCATTCCAGGATGGCAGATGGGACACAAGACATAATAGAACTGAGGCTAAGGGTATATATTGTTTTTTTCTTATTTATCATCAAT
TATTTTCACCTTCAGTTTCTTGATGACTTTGGATATCACACTGGGTCCCACCGCAGTGTGGTTTTCAGTAAATATACTTTATTAAAAG
CTGAAGTGCTAGCCCAAAGTAAGGAGATAAAACAGACTACACGATATCTAGGCTCTATGTAGTGAGGAGCCCCAGGGTTTCACTT
AAAAATCTTCTCCCATAAAGTTGACCAGACTTGATATTTTTTAGCCTGTGATGGGATAAGACCACACCTCAGGGCTCTCCCTGAGAG

AAAGCCTGCAGACAGGTGGTGCCTTGATCTGTATGATGCAATCTGCAGATGGGGCATCCCTCTTCCTTCTGTGGCATAATTCCTAC
GGACAGCTTAACATGGGGATTTAATAAAAATGTACATATTTTCTGTGAAGAATCCAACAACAAATGAAAGAAGTTTTTGCCTGCCT
TTGAAATGGGTGGACCCTGAAACATTGGTCTGTTTGAAGTGGCATCACAACCCATACACAGATGCTTCATTCCTGTCGGCCATTACT
GGACTGGACTAGAACACTAAGTCCTGATAAGACACATCATTTGCAATGAGAATATATTCTGTTTGTAAGCCTTTCTGATGTGCCATT
ATTGCTGCTGTGTTTCCTGTTTTATGTAGGTGGTAGCCCTCGGAGAGGTACCAGATGGGACTGTGGTTACTGTCATGGCGGGTAAC
GATGAAAATTATTCTGCTGAGCTCCGGAATGCCTCTGCTGTTATGAAAAACCAAGTAGCAAGGTTCAACGATCTGAGATTTGTGGG
CCGGAGTGGACGAGGTAGGTCTCTGACTTTTGATACTGATAATAGAATAAGCACATTAGGCTCCTTTGATGAAATGTAGACTAGTC
TGTATACAAATCAGCACCTTCTTTTTCTGAATAGAATTACTGAAGATTTGATTTAAATACATCCAGATGAAGTTGAGTGTTTTTCTGA
GTACTCAGGCCTTTTTCATTTATTTTATGATATTGAAAATTCAAACAATGTTTTCAGAAAAACTAGCTCCTAATTTCACTGGGTGTGA
GCATATAAGGTAGAGAAAAGAACAGTTGGAGGATCAACTTCTGAATTATGAGGAGTCAAATAACTGAGTAAATTGAGTGGTAAA
GGGGAAAGAAAAATGGATATGTTGGATCAATTTCCTGGTAATATCAATTTTGGTCTAAGTTGTAGCAAGGACAGCTGGCATAATG
CTCTTCACTTATCACAGGTTACCTCTATATGATTTAATTTACTCTCATAGTAAGAATTTATTAAAAACCATGGTTTTTTTTAACATAA
GGCTTTTCATTTGAATAAGTGAATAATGTATTACTTATGTTAGAGTGACTATGTAAGGTTGCCTAGACAGATGAATGATTAGTACTC
TAATATTAATAGTTCCATACTATGTAAGGATTCCCATCATCTGAATCCATATTCAAAAATTAGAGATCCTTAAATAGAAAATTATCCC
TTTTAGGGTTTTTGTAACTGTTTGATAAATGAGGTTAATGTTTTTTAAAAAAGTCTTTAAGGAGGCCAATATTTTGGCTTTTATTTAC
TTATTCTACAGAAAGACTTTTACTAGTTACTCACTATTAGAAGCATTATGCTTATTTATTTACTTAACTGAATCTAATATTTCCATTTTA
AGTATTTTGATAAGAAGAAAGAAAATAGACCACTTTAGAACTGAGGTGAAATAAAGCCTTATGGAATCATTGCAACCCAGGTTTG
AATAGGAAATGAGGAGCACCACTTTTTAAACCATGTCTAATAATTGCTCTTTAGTTTGGATCAGATGTCAAATTATTACCATTGTAA
AAACTTGATAAGGGAAATTCAAGCAAGGCTGTTTCCGATGAGTAGTGTTAGTTTACTTTCTAGGAACAGGGCCAAGAAGTTGTACT
TTTTAACTTAAAAAAAAATGATTCATGCTAATGAGATACTG

4G7 clone genomic sequence (insertion in green)

GAGGGCATCTGGAAGCTGAATGAGGAAGACATGGAAATAATCTGAATATAGAGTCCTGGAAGTTCGTTGAGAGAAGGGATTGTT
ACCAAGGGGCAGGATCTTGGCAATGGATTAGGAGGAGTGTTTCATATATGACAGTGAAGGATCTGCACCAGCATCTCCTTACTGG
GCTCTGTGTTAATCAAAGGCACACATTTGCATGTTAGCTGACTCAGGTTAAAGTGTTATCTCTAGTCTTAGAGCAGAGAAAATGTTT
GTTTGTTCCAAAGATGGATTAATCTAGATCTTTTCTTGATTGAGTTAATTTGCTTCAGATTAACTGTTGAAGATTAAGCAGTCAAGTA
TGTCATTCCAGGATGGCAGATGGGACACAAGACATAATAGAACTGAGGCTAAGGGTATATATTGTTTTTTTCTTATTTATCATCAAT
TATTTTCACCTTCAGTTTCTTGATGACTTTGGATATCACACTGGGTCCCACCGCAGTGTGGTTTTCAGTAAATATACTTTATTAAAAG
CTGAAGTGCTAGCCCAAAGTAAGGAGATAAAACAGACTACACGATATCTAGGCTCTATGTAGTGAGGAGCCCCAGGGTTTCACTT
AAAAATCTTCTCCCATAAAGTTGACCAGACTTGATATTTTTTAGCCTGTGATGGGATAAGACCACACCTCAGGGCTCTCCCTGAGAG
AAAGCCTGCAGACAGGTGGTGCCTTGATCTGTATGATGCAATCTGCAGATGGGGCATCCCTCTTCCTTCTGTGGCATAATTCCTAC
GGACAGCTTAACATGGGGATTTAATAAAAATGTACATATTTTCTGTGAAGAATCCAACAACAAATGAAAGAAGTTTTTGCCTGCCT
TTGAAATGGGTGGACCCTGAAACATTGGTCTGTTTGAAGTGGCATCACAACCCATACACAGATGCTTCATTCCTGTCGGCCATTACT
GGACTGGACTAGAACACTAAGTCCTGATAAGACACATCATTTGCAATGAGAATATATTCTGTTTGTAAGCCTTTCTGATGTGCCATT
ATTGCTGCTGTGTTTCCTGTTTTATGTAGGTGGTAGCCCTCCATCTGGTGGAGAGGTACCAGATGGGACTGTGGTTACTGTCATGG
CGGGTAACGATGAAAATTATTCTGCTGAGCTCCGGAATGCCTCTGCTGTTATGAAAAACCAAGTAGCAAGGTTCAACGATCTGAGA
TTTGTGGGCCGGAGTGGACGAGGTAGGTCTCTGACTTTTGATACTGATAATAGAATAAGCACATTAGGCTCCTTTGATGAAATGTA
GACTAGTCTGTATACAAATCAGCACCTTCTTTTTCTGAATAGAATTACTGAAGATTTGATTTAAATACATCCAGATGAAGTTGAGTG
TTTTTCTGAGTACTCAGGCCTTTTTCATTTATTTTATGATATTGAAAATTCAAACAATGTTTTCAGAAAAACTAGCTCCTAATTTCACT
GGGTGTGAGCATATAAGGTAGAGAAAAGAACAGTTGGAGGATCAACTTCTGAATTATGAGGAGTCAAATAACTGAGTAAATTGA
GTGGTAAAGGGGAAAGAAAAATGGATATGTTGGATCAATTTCCTGGTAATATCAATTTTGGTCTAAGTTGTAGCAAGGACAGCTG
GCATAATGCTCTTCACTTATCACAGGTTACCTCTATATGATTTAATTTACTCTCATAGTAAGAATTTATTAAAAACCATGGTTTTTTTT
TAACATAAGGCTTTTCATTTGAATAAGTGAATAATGTATTACTTATGTTAGAGTGACTATGTAAGGTTGCCTAGACAGATGAATGAT
TAGTACTCTAATATTAATAGTTCCATACTATGTAAGGATTCCCATCATCTGAATCCATATTCAAAAATTAGAGATCCTTAAATAGAAA
ATTATCCCTTTTAGGGTTTTTGTAACTGTTTGATAAATGAGGTTAATGTTTTTTAAAAAAGTCTTTAAGGAGGCCAATATTTTGGCTT
TTATTTACTTATTCTACAGAAAGACTTTTACTAGTTACTCACTATTAGAAGCATTATGCTTATTTATTTACTTAACTGAATCTAATATTT
CCATTTTAAGTATTTTGATAAGAAGAAAGAAAATAGACCACTTTAGAACTGAGGTGAAATAAAGCCTTATGGAATCATTGCAACCC
AGGTTTGAATAGGAAATGAGGAGCACCACTTTTTAAACCATGTCTAATAATTGCTCTTTAGTTTGGATCAGATGTCAAATTATTACC
ATTGTAAAAACTTGATAAGGGAAATTCAAGCAAGGCTGTTTCCGATGAGTAGTGTTAGTTTACTTTCTAGGAACAGGGCCAAGAA
GTTGTACTTTTTAACTTAAAAAAAAATGATTCATGCTAATGAGATACTG
